# Adolescent to adulthood weight trajectories and the risk of obesity-related cancers, overall and early-onset: a population-based cohort study

**DOI:** 10.1016/j.eclinm.2025.103711

**Published:** 2026-01-05

**Authors:** Cole D. Bendor, Aya Bardugo, Avishai M. Tsur, Estela Derazne, Itay I. Shemesh, Lotmit Bourvine, Dror Dicker, Ben Boursi, Amir Tirosh, Arnon Afek, Ran S. Rotem, Gabriel Chodick, Gilad Twig

**Affiliations:** a Department of Military Medicine, Hebrew University, Jerusalem, Israel; b Israel Defense Forces Medical Corps, Ramat Gan, Israel; c The Gertner Institute for Epidemiology & Health Policy Research, Sheba Medical Center, Tel Hashomer, Ramat Gan, Israel; d Department of Preventive Medicine and Epidemiology, School of Public Health, Gray Faculty of Medical & Health Sciences, Tel Aviv University, Tel Aviv, Israel; e The Dina Recanati School of Medicine, Reichman University, Herzliyya, Israel; f Department of Internal Medicine B, Sheba Medical Center, Tel Hashomer, Ramat Gan, Israel; g Faculty of Medicine, Tel Aviv University, Tel Aviv, Israel; h Novo Nordisk, Kfar Saba, Israel; i Novo Nordisk Health Care AG, Zürich, Switzerland; j Rabin Medical Center, Hasharon Hospital, Department of Internal Medicine D and Obesity Clinic, Petah Tikva, Israel; k Institute of Oncology, Sheba Medical Center, Ramat Gan, Israel; l Center for Clinical Epidemiology and Biostatistics, University of Pennsylvania, Philadelphia, PA, USA; m Division of Endocrinology, Diabetes and Metabolism, Sheba Medical Center, Tel Hashomer, Ramat Gan, Israel; n Maccabitech, Maccabi Healthcare Services, Tel Aviv, Israel; o Department of Environmental Health, Harvard T.H. Chan School of Public Health, Boston, MA, USA

**Keywords:** Adolescents, Obesity, Cancer, Malignancy, BMI trajectory, Weight loss

## Abstract

**Background:**

High body mass index (BMI) is a modifiable cancer risk factor, projected to surpass smoking as the leading preventable risk factor. The impact of weight change from late adolescence to adulthood on cancer risk remains unclear. We aimed to assess the association between adolescence-to-adulthood BMI trajectories and obesity-related cancer risk.

**Methods:**

A population-based cohort study of 800,024 people (45.1% women) insured by a large state-mandated health provider. BMI was measured during military pre-recruitment evaluations during 1967–2018 in adolescence and in subsequent clinic visits in adulthood during 1998–2020. Follow-up began one year after an adult BMI measurement until cancer diagnosis, death, transfer to another health provider, or December 16, 2021. BMI trajectories from adolescence to adulthood were classified as lean-to-lean, lean-to-high, high-to-lean, and high-to-high (cutoff: sex-specific and age-specific 85th percentile in adolescence, defined according to the United States Centers for Disease Control and Prevention growth charts, and 25 kg/m^2^ in adulthood). Weight change was also assessed per 5% increments. The primary outcome was obesity-related cancers including esophagus, postmenopausal breast, liver and gallbladder, stomach, pancreas, colon and rectum, kidney, multiple myeloma, thyroid, uterus and ovary. The secondary outcome was obesity-related cancers diagnosed before age 50 years (early-onset cancers). Cox proportional hazards models were applied.

**Findings:**

During 7,610,263 person-years, 6,376 people were diagnosed with obesity-related cancers, at a mean age of 53.3 ± 9.8 years. Adjusted hazard ratios (HRs) were 1.31 (95% confidence interval [CI], 1.24–1.39) for lean-to-high, 1.01 (95% CI, 0.78–1.31) for high-to-lean, and 1.47 (95% CI, 1.34–1.61) for high-to-high groups, compared to the lean-to-lean group. Respective HRs for early-onset obesity-related cancers were 1.33 (95% CI, 1.20–1.47), 0.88 (95% CI, 0.60–1.31), and 1.39 (95% CI, 1.20–1.61). Each 5% weight gain conferred a 3% increased hazard (95% CI, 1.02–1.03), with a similar 3% increase for early-onset cancers (95% CI, 1.02–1.04). Cancer-specific risks included 3% (95% CI, 1.02–1.04) for postmenopausal breast cancer, 3% (95% CI, 1.01–1.04) for colorectal cancer, 4% (95% CI, 1.02–1.05) for thyroid cancer, 5% (95% CI, 1.04–1.07) for kidney cancer, and 8% (95% CI, 1.06–1.09) for uterine cancer. Some cancers, including leukemia and non-Hodgkin's lymphoma, were not associated with weight gain but were positively associated with high adolescent BMI.

**Interpretation:**

Maintaining a healthy BMI from adolescence to adulthood may reduce obesity-related cancer risk, including early-onset, highlighting the importance of early weight management strategies.

**Funding:**

10.13039/501100004191Novo Nordisk, Israel.

**Research in context**

**Evidence before this study**

We searched PubMed from January 1, 2000, and January 1, 2025, for studies evaluating the association between weight status trajectories and cancer risk. We used the search terms “BMI” OR “body mass index” OR “weight” OR “obesity” AND “trajectory” OR “change” OR “trend” AND “cancer” OR “malignancy”. Most studies evaluated body mass index (BMI) at mid-adulthood, often close to diagnosis, and did not capture weight change from early life. Fewer studies have examined weight change from age 18 or 20 to midlife. The potential mitigating effects of weight normalization in adulthood after adolescent obesity remain unknown. We found no population-based studies assessing the risk for early-onset cancers diagnosed before age 50 years.

**Added value of this study**

In this nationwide cohort of over 800,000 individuals with measured BMI in adolescence and adulthood, we show that people who gained weight from a low to normal status in adolescence to overweight or obesity in adulthood were at increased risk of developing obesity-related cancers, including early-onset cancers. Importantly, individuals with obesity in adolescence who normalized their weight in adulthood had no excess cancer risk compared to those who remained lean. The results were consistent across multiple cancer types and robust to a wide range of sensitivity analyses. Each 5% weight gain conferred a 3% increased risk of all obesity-related cancers and early-onset obesity-related cancers.

**Implications of all the available evidence**

Our findings highlight the importance of early-life weight trajectories in shaping future cancer risk. Public health strategies should focus not only on preventing obesity in adolescence, but also on identifying opportunities for weight normalization in early adulthood. The results support early adulthood as a critical window for interventions to reduce the burden of obesity-related and early-onset cancers. These findings may inform clinical guidelines and public health policies aimed at cancer prevention across the life course.

## Introduction

Cancer is a leading cause of morbidity and mortality worldwide, and utilizes high healthcare resources.^1^^,^^2^ The United Nations Sustainable Development Goals emphasized reducing cancer burden through addressing modifiable risk factors, especially among young adults.^2^ High BMI, the third leading modifiable risk factor for global cancer burden,^3^ is expected to surpass smoking as the leading preventable risk factor.^4^^,^^5^ High BMI is causally related to at least 12 malignant cancer types,^6^^,^^7^ which together account for nearly 40% of all incident cancers diagnosed in the United States.^7^

Incidences of early-onset cancers (diagnosed before age 50 years), and particularly obesity-related cancers, have increased over the past three decades.^8^ In the United States, the annual percentage change in age-standardized incidence rate between 2010 and 2019 was 1.00% for early-onset obesity-related cancers and 0.28% for all early-onset cancers, contrasting with a 0.87% decline in cancers diagnosed after age 50.^9^ Most studies that assessed obesity and cancer relied on BMI measurements taken at mid-adulthood, often a few years before cancer diagnosis.^10^, ^11^, ^12^ In our nationwide cohort study of 2.3 million adolescents, adolescent obesity was associated with cancer diagnosed in young adulthood.^13^ While the association between high BMI and cancer risk later in life is well-documented, the impact of BMI trajectories from adolescence to adulthood on early-onset cancer remains understudied. It is unclear whether weight reduction during young adulthood could mitigate this risk. In a population-based cohort of 800,024 people, we explored the association between adolescent-to-adulthood BMI trajectories and obesity-related cancers.

## Methods

### Study population

**Fig. 1:**
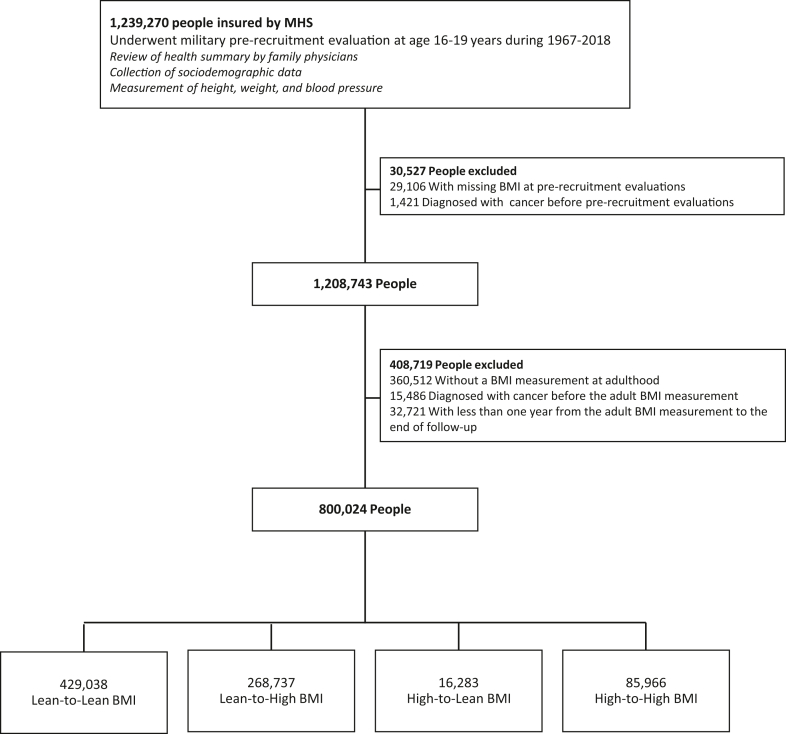
**Study cohort build-up**. The study groups included (1) those with lean adolescent body mass index (BMI) and lean adulthood BMI (lean-to-lean BMI; the reference group); (2) lean adolescent BMI and high adulthood BMI (lean-to-high BMI); (3) high adolescent BMI and lean adulthood BMI (high-to-lean BMI); (4) high adolescent BMI and high adulthood BMI (high-to-high BMI). MHS, Maccabi Health Services.

For this population-based cohort study, data were obtained from the national Israeli conscription database and linked to Maccabi Healthcare Services (MHS), Israel's second-largest state-mandated health provider, which insures about one-quarter of the Israeli population. Included were people with BMI data collected during two periods: in adolescence (age 16–19 years) as part of pre-recruitment evaluations during 1967–2018, one year before mandatory military service, and in adulthood at routine MHS clinic visits during 1998–2020. Exclusion criteria were missing weight or height data during adolescence or adulthood, and cancer diagnoses prior to these measurements. To minimize bias from cancer-associated weight loss,^14^ we excluded those with less than one year follow-up after the adult BMI measurement (Fig. 1).

### Adolescent evaluation

During the study period, Israeli adolescents underwent comprehensive evaluations in the year preceding mandatory military service, including detailed interviews, health record reviews, physical examinations, and cognitive assessments.^15^^,^^16^ Individuals with a positive medical history or abnormal findings underwent additional tests or consultations with board-certified specialists to confirm diagnoses and determine medical fitness. Weight and height were measured with light clothing using a beam balance and stadiometer, systematically documented since 1967.^17^ BMI was calculated as weight in kilograms divided by height in meters squared. BMI values were classified according to sex-specific and age-specific percentiles defined by the US Center of Disease Control and Prevention criteria, validated for the Israeli population,^18^ categorized into: lean adolescent (<85th percentile) and high adolescent (≥85th percentile). Residential socioeconomic position was based on the scoring system (on a 1–10 scale) of the Israeli Central Bureau of Statistics for local authorities,^19^ and categorized as low (1–4), medium (5–7), and high (8–10).^20^ Additional sociodemographic data included years of education (<11 or ≥11 years), according to the Israeli Ministry of Education^21^; country of birth (Israel or elsewhere); and country of origin classified by the examinee's father's country of birth, or by the grandfather's country of birth if the father was born in Israel.^16^ As part of the pre-recruitment evaluation, conscripts underwent a general intelligence test that was administered by trained personnel. As described previously, the test included four intellectual domains, and the overall score (on a nine-point scale) was found to be highly correlated with the Weschler Adult Intelligence Scale IQ.^22^ Scores were categorized as low (1–3), medium (4–7), and high (8–9).

### MHS clinical data

Under Israel's National Health Insurance Law, membership in one of four state-mandated health providers is compulsory for all Israeli citizens. The health providers, including MHS, cannot bar any Israeli citizen who wishes to enroll, representing all sections of the Israeli population.^23^ The first BMI measurement recorded at a primary care visit in adulthood (at age of 20 years or older) was considered. Digital documentation of BMI measurements was established in 1998, and reached about 85% in 2010.^24^ Using cut-off points established by the US Center of Disease Control and Prevention, BMI values were categorized to: lean adulthood BMI (BMI <25 kg/m^2^) and high adulthood BMI (BMI ≥25 kg/m^2^). Smoking status was obtained from the MHS database, categorized as never smokers or ever smokers (current or past).^21^^,^^23^

### Diabetes diagnosis

Diabetes status in adulthood was determined from the MHS diabetes registry based on the following criteria: Serum glucose concentrations of ≥200 mg/dL in at least two tests conducted at least one month apart; at least two purchases of glucose-lowering medications within three months, and at least one fasting plasma glucose of ≥126 mg/dL (7.0 mmol/L) or glycated hemoglobin (HbA1c) of ≥6.5% (48 mmol/mol); at least two separate insulin purchases within three months, followed by ongoing purchases; a diagnostic code assigned by a board-certified physician, with HbA1c ≥ 6.5% or fasting plasma glucose of ≥126 mg/dL in two tests six months before or after diagnosis; HbA1c ≥ 7.25% (55 mmol/mol). Individuals who met one or more of these criteria were flagged for review by primary care physicians, who were required to actively document the diagnosis of diabetes.^23^

### MHS cancer registry

The MHS cancer registry routinely collects personal-level information on diagnosed incident cancers among its members from the Israel National Cancer Registry (INCR). The data includes cancer diagnoses, classifications, and diagnoses dates, obtained from hospital discharge records, pathology and cytology reports, oncology and hematology institutes, and death certificates. The INCR achieves a case completeness of 94% for all diagnosed cancers.^25^ To enhance accuracy, the MHS cancer registry supplements its data by linking with records from the MHS drug approval committee.

### Adolescent-to-adulthood BMI trajectory groups

The four study groups were: lean adolescent BMI and lean adulthood BMI (lean-to-lean BMI); lean adolescent and high adulthood BMI (lean-to-high BMI); high adolescent and lean adulthood BMI (high-to-lean BMI); and high adolescent and high adulthood BMI (high-to-high BMI). Percent weight change was also assessed as a continuous variable ($\frac{adulthood\;weight\;-\;adolescent\;weight}{adolescent\;weight}*100$, per 5 unit change).

### Outcome defintion

The primary outcome was the incidence of obesity-related cancers, as recorded by the MHS cancer registry. Obesity-related cancers were determined according to the International Agency for Research on Cancer,^6^ and encompassed 11 cancers: esophagus, postmenopausal breast, liver and gallbladder, stomach, pancreas, colon and rectum, kidney, multiple myeloma, thyroid, uterus, and ovary. Each cancer was also evaluated separately. Follow-up extended from one year after the adult BMI measurement until cancer diagnosis, death, transfer to another state-mandated health provider, or December 16, 2021. The secondary outcome was obesity-related cancers diagnosed before age 50 years.

### Ethics

The Institutional Review Boards of the Israel Defense Forces Medical Corps and MHS approved this study and waived the requirement for informed consent based on stringent maintenance of study member anonymity.

### Statistical analysis

Age at adolescent evaluation and at adult BMI measurement were treated as continuous variables; other variables were categorical. Means and standard deviations (SD) were reported for continuous variables, and numbers and percentages for categorical variables. Incidence rates of cancers were calculated per person-years. Cox proportional hazard models were applied to estimate unadjusted hazard ratios (HRs) and 95% confidence intervals (CIs) for incident cancer in the lean-to-high, high-to-lean, and high-to-high BMI groups; lean-to-lean BMI served as the reference group. Kaplan–Meier survival curves were plotted to depict the cumulative incidences of obesity-related cancers using age as the timescale. The proportional hazards assumption was tested graphically with the use of log-minus-log plots. Linearity of the continuous variables (age and year of BMI measurement) was assessed with Martingale residual plots. The 95% CIs for hazard ratios were computed using the Wald method derived from the Cox proportional hazards model. CIs for crude incidence rates were calculated based on the Poisson distribution. Covariates were predetermined based on prior knowledge and previous studies from our group evaluating adolescent BMI and long-term cardiometabolic or cancer risk.^13^ Minimally adjusted models include sex and age at the adult BMI measurement. The model was further adjusted for the year of the adult BMI measurement, education, residential socioeconomic position, cognitive performance, and birth country as previously described.^13^ 16,004 (2.0%) people with missing data were excluded from adjusted analyses. Penalized spline models with 4 degrees of freedom were used to depict hazard functions (and 95% CIs) for obesity-related cancers and for each cancer type, across the entire continuum of adolescent-to-adulthood percent weight change and delta BMI. This analysis was conducted using R version 4.3.1 (R Foundation for Statistical Computing). The same adjusted model was applied to continuous analyses, adding adolescent BMI. Analyses used IBM-SPSS version 29, unless otherwise mentioned.

### Subgroups and sensitivity analyses

We performed several subgroup and sensitivity analyses. First, to account for selection bias caused by excluding those without adulthood measurement, the Cox modeling was applied to compare HRs of adolescent BMI and incident cancer between those with and without an adult BMI measurement. Second, to account for possible inter-group differences in the ages of adult BMI measurements and the interval between BMI measurements, several sensitivity analyses were performed: Analysis was stratified by the median age of the adult weight measurement and by the period of birth; the study period was limited to the years that BMI measurements were fully digitalized; analyses were stratified by age at adult BMI measurement (<30 years, 30–39 years, 40–49 years, and ≥50 years); a matched analysis was conducted, using the high-to-lean BMI group as the reference. Each participant in this group was matched (1:3 ratio) to individuals from the other trajectory groups based on sex, the elapsed time between adolescent and adult BMI measurements (±1 year) and age at the adult BMI measurement (±1 year). Third, Individuals with two adult BMI measurements were included, to confirm the adult measurement and reduce misclassification in the BMI trajectory. Fourth, to evaluate potential bias from missing covariate data, we included an indicator for missing data in the model and repeated the analyses separately for participants with and without missing values. Fifth, the analysis was stratified by sex. Sixth, to mitigate confounding by coexisting illness, the analysis was limited to those with unimpaired health at adolescence (lack of any history of chronic medical treatment, the need for a medical follow-up, or a history of major surgery) and by adjusting for smoking and diabetes status throughout the study period. Seventh, people who underwent bariatric surgery were excluded, as this could decrease obesity-related cancer incidence.^26^ Eighth, given evidence linking high BMI with hematological cancers,^11^^,^^27^ people with Hodgkin's lymphoma, non-Hodgkin's lymphoma, and leukemia were additionally included in the outcome. Ninth, the outcome was set as obesity-related cancers diagnosed before age 40 years. Tenth, the high-to-lean group was compared to an adolescent BMI-matched cohort, as BMI at adolescence in the high-to-lean group may have been lower than in the high-to-high group. Eleventh, to assess a potential dose–response relationship within the high BMI category, we stratified adolescent and adulthood BMI into overweight (85th–95th percentile in adolescence and BMI of 25–30 kg/m^2^ in adulthood) and obesity (≥95th percentile in adolescence and BMI of ≥30 kg/m^2^ in adulthood). Twelfth, the model was repeated with follow-up starting at the first (adolescent) BMI measurement, treating this time point as the hypothetical intervention (time zero).^28^ Thirteenth, to distinguish whether cancer risk was primarily related to early-life adiposity or weight gain thereafter, we performed an additional continuous analysis in which both adolescent BMI and the adolescent-to-adulthood change in BMI (each per 1 kg/m^2^ increment) were entered simultaneously as continuous covariates within the same fully adjusted Cox proportional hazards model used in the main analyses. Fourteenth, our findings were compared directly with Zheng et al.’s study,^29^ using a continuous 5 kg weight difference as the exposure. Subgroup and sensitivity analyses were considered exploratory; therefore, no formal multiplicity adjustment was performed.

### Role of funding source

The study was funded by Novo Nordisk. The funder had no role in the study design; data collection and analysis; writing of the report; or the decision to submit the manuscript for publication. Two co-authors (I.I.S. and L.B.), who are employees of Novo Nordisk, reviewed and edited earlier manuscript versions as part of the authorship process but were not involved in the study design or data handling. The corresponding author made the final decision to submit the manuscript for publication.

## Results

### Cohort build-up

Of the 1,208,743 people who completed a BMI measurement at adolescence, 360,512 (30%) did not undergo a BMI measurement in adulthood (Fig. 1). Sociodemographic characteristics were similar, although those without adult BMI data were more likely to enter the study in later calendar years and thus younger at the end of follow-up than were those with an adult BMI measurement (mean [SD] 27.7 [9.2] versus 44.1 [12.5] years, P < 0.0001, Supplementary Table S1). The distributive incident cancers reflected a lower proportion of cancers typical at older ages, such as postmenopausal breast cancers (Supplementary Figure S1). High adolescent BMI was associated with a similarly increased risk for obesity-related cancers, among those with and without adult BMI measurements: HRs of 1.32 (95% CI, 1.22–1.43) and 1.42 (95% CI, 1.12–1.81) respectively (Supplementary Table S2).

### Characteristics of the study cohort

Table 1 and Supplementary Table S3 present the baseline characteristics of the 800,024 people included in the study, according to BMI trajectory groups. The median age of the adult BMI measurements was 33.1 years (interquartile range 24.7–42.2), differed between the groups, and was highest for the lean-to-high group. The lean-to-lean, lean-to-high, high-to-lean, and high-to-high BMI groups constituted 53.6%, 33.6%, 2.0%, and 10.7%, respectively, of the cohort. For these respective groups, the mean (SD) changes in weight from adolescence to adulthood were 6.3 (8.0), 21.5 (11.2), −9.6 (10.5), and 11.3 (14.7) kilograms. The respective mean percentages of weight change were +11.6%, +35.1%, −11.8%, and +14.3%.

**Table 1:** Characteristics of adolescent-adulthood BMI trajectory groups.

	Lean-to-Lean BMI	Lean-to-High BMI	High-to-Lean BMI	High-to-High BMI	Total
Number of people (%)	429,038 (53.6)	268,737 (33.6)	16,283 (2.0)	85,966 (10.7)	800,024
Men, number (%)	197,733 (46.1)	182,309 (67.8)	7415 (45.5)	51,430 (59.8)	438,887 (54.9)
Women, number (%)	231,305 (53.9)	86,428 (32.2)	8868 (54.5)	34,536 (40.2)	361,137 (45.1)
**At late adolescence**					
Age at evaluation, years Mean ± S.D	17.3 ± 0.4	17.4 ± 0.4	17.2 ± 0.4	17.3 ± 0.5	17.3 ± 0.4
Median [IQR]	17.2 [17.0; 17.4]	17.3 [17.1; 17.5]	17.2 [17.0; 17.4]	17.2 [17.0; 17.5]	17.2 [17.1; 17.5]
BMI, kg/m^2^					
Mean ± S.D	20.2 ± 2.0	21.7 ± 2.0	27.4 ± 2.7	28.6 ± 3.2	21.7 ± 3.4
Median [IQR]	20.1 [18.7; 21.5]	21.7 [20.2; 23.2]	26.6 [25.8; 28.1]	27.7 [26.2; 30.1]	21.1 [19.4; 23.2]
Residential socioeconomic position, %					
Low	17	20	17	21	18
Medium	51	51	53	54	52
High	32	28	30	25	30
Intelligence score, %					
Low	10	11	13	16	11
Medium	72	71	74	71	72
High	17	18	14	13	17
Graduated high school, %	94	86	95	91	91
Born in Israel, %	82	82	84	82	82
Country of origin^a^					
Israel	43,669 (10)	17,410 (7)	2087 (13)	9465 (11)	72,631 (9)
Former USSR	77,416 (18)	39,737 (15)	3089 (19)	16,967 (20)	137,209 (17)
Asia	93,687 (22)	62,348 (23)	3082 (19)	15,864 (19)	174,981 (22)
Africa	76,076 (18)	58,070 (22)	3146 (19)	16,441 (19)	153,733 (19)
Europe and north America	133,771 (31)	88,381 (33)	4732 (29)	26,414 (31)	253,298 (32)
Ethiopia	1641 (<1)	442 (<1)	41 (<1)	144 (<1)	2268 (<1)
Unimpaired health^a^, number (%)	355,643 (83)	230,332 (86)	12,861 (79)	67,912 (79)	666,748 (83)
**At adulthood**					
Age at adult measurement of BMI					
Mean ± S.D	32.3 ± 9.9	39.5 ± 10.3	29.3 ± 8.6	31.4 ± 9.8	34.6 ± 10.6
Median [IQR]	29.8 [23.5; 38.9]	39.3 [31.7; 47.3]	25.8 [22.6; 33.9]	28.6 [22.9; 37.4]	33.1 [24.7; 42.2]
Adult BMI, kg/m^2^					
Mean ± S.D	21.8 ± 2.1	28.5 ± 3.3	23.1 ± 1.6	31.8 ± 5.0	25.1 ± 4.8
Median [IQR]	22.0 [20.3; 23.4]	27.7 [26.2; 29.9]	23.5 [22.3; 24.3]	30.8 [28.0; 34.6]	24.4 [21.7; 27.7]
Delta weight					
kg, Mean ± S.D	6.3 ± 8.0	21.5 ± 11.2	−9.6 ± 10.5	11.3 ± 14.7	11.7 ± 12.6
Percent, %					
Mean ± S.D	11.6 ± 14.8	35.1 ± 19.2	−11.8 ± 11.9	14.3 ± 18.0	19.3 ± 20.4
Median [IQR]	10.0 [2.0; 19.0]	32.8 [22.4; 44.7]	−10.8 [−17.3; −5.3]	11.9 [2.5; 23.6]	16.4 [5.5; 30.4]
Delta BMI, kg/m^2^ Mean ± S.D	1.6 ± 2.3	6.8 ± 3.4	−4.3 ± 3.2	3.2 ± 4.8	3.4 ± 4.0
Diagnosed with diabetes before cancer, number (%)	6222 (1)	29,828 (11)	238 (1)	8906 (10)	45,194 (6)
Smoking, number (%)	165,930 (39)	107,601 (40)	7568 (47)	37,846 (45)	318,945 (40)

Sociodemographic and body mass index (BMI) data at late adolescence were extracted from the Israel Defense Forces military pre-recruitment dataset. BMI and clinical data at adulthood were retrieved from the Maccabi Healthcare Service dataset. BMI was classified according to sex-specific and age-specific percentiles defined by United States Centers for Disease Control and Prevention. The two adolescent categories were: lean (BMI <85th percentile) and high (BMI ≥85th percentile). The two adult categories were: lean (BMI <25 kg/m^2^) and high (BMI ≥25 kg/m^2^). The four study groups included those with: (1) lean adolescent BMI and lean adulthood BMI (lean-to-lean BMI; the reference group); (2) lean adolescent BMI and high adulthood BMI (lean-to-high BMI); (3) high adolescent BMI and lean adulthood BMI (high-to-lean BMI); (4) high adolescent BMI and high adulthood BMI (high-to-high BMI). Country of origin was classified by the examinee's father's country of birth or by the grandfather's country of birth if the father was born in Israel.

Data were missing from 7570 (0.9%) people for intelligence score, from 3193 (0.4%) people for education, from 10,674 (1.3%) people for residential socioeconomic position, from 332 (<0.1%) people for country of birth, from 5904 (0.7%) of country of origin and from 6298 (0.8%) people for smoking status.

S.D, standard deviation; IQR, interquartile range.

a Unimpaired health was defined for people without any morbidities at pre-recruitment evaluation at adolescence that required medical follow-up or chronic treatment, and without a history of cancer or major surgery.

### BMI trajectories and obesity-related cancers

**Fig. 2:**
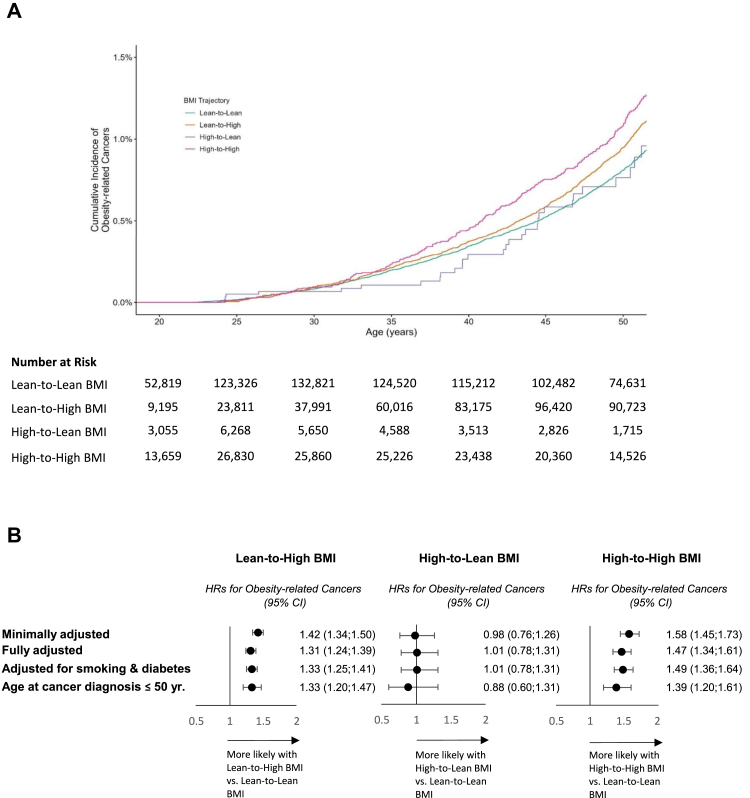
**Adolescent-to-adulthood BMI trajectory and the risk for obesity-related cancers. A.** Kaplan–Meier survival curves are shown for obesity-related cancers among people with lean-to-lean body mass index (BMI), lean-to-high BMI, high-to-lean BMI, and high-to-high BMI. The population at risk is presented for each interval. ∗P-value for linear trend <0.05. **B**. Hazard ratios (HRs) for obesity-related cancers among people with lean-to-high BMI, high-to-lean BMI, and high-to-high BMI, compared to people with lean-to-lean BMI. The minimally adjusted model includes sex and age at the adult BMI measurement (6376 incident cases out of 800,024). The adjusted model also includes the year of the adult BMI measurement, education, intelligence score, residential socioeconomic position, and birth country (5972 incident cases out of 784,020). The model was further adjusted for smoking and diabetes status (5970 incident cases out of 777,806). The adjusted model was applied for the analysis of cancer diagnosis at age ≤50 years (1854 incident cases out of 705,931). The definition of obesity-related cancers was determined according to the International Agency for Research on Cancer (IARC),^6^ encompassing 11 malignant cancers: esophagus, postmenopausal breast, liver and gallbladder, stomach, pancreas, colon and rectum, kidney, multiple myeloma, thyroid, uterus, and ovary.

During a cumulative follow-up of 7,610,263 person-years, 6376 people were diagnosed with obesity-related cancers at a mean age (SD) of 53.3 (9.8) years; 1886 (30%) were diagnosed before the age of 50 years. The characteristics and distributions of malignant cancer types (including those not obesity-related) are presented in Supplementary Tables S4 and S5. The median follow-up from the adult BMI measurements was 9.7 years (interquartile range 5.8–13.3, Table 2). The crude incidence rates for obesity-related cancers (95% CI, per 10,000 person-years) were 6.1 (5.9–6.4), 11.8 (11.4–12.3), 4.6 (3.6–6.0), and 7.9 (7.3–8.6) among the lean-to-lean, lean-to-high, high-to-lean, and high-to-high BMI groups, respectively (Table 2). Kaplan–Meier survival curves are presented in Fig. 2A. The minimally adjusted HRs (sex and age at adult BMI measurement) for the three latter groups were 1.42 (95% CI, 1.34–1.50), 0.98 (95% CI, 0.76–1.26), and 1.58 (95% CI, 1.45–1.73), respectively, compared to the lean-to-lean group (Fig. 2B). Point estimates were similar following further adjustment for sociodemographic variables; HRs were 1.31 (95% CI, 1.24–1.39), 1.01 (95% CI, 0.78–1.31), and 1.47 (95% CI, 1.34–1.61), respectively (Fig. 2B).

**Table 2:** Associations between adolescence-to-adulthood BMI trajectory groups and obesity-related cancers.

	Lean-to-Lean BMI	Lean-to-High BMI	High-to-Lean BMI	High-to-High BMI	Total
Number of people	429,038	268,737	16,283	85,966	800,024
**Obesity-related cancers**					
Number of incident cancers	2368	3315	62	631	6376
Follow-up					
Mean ± S.D	9.0 ± 4.3	10.4 ± 4.6	8.2 ± 4.4	9.2 ± 5.0	9.5 ± 4.5
Median [IQR]	9.2 [5.5; 12.7]	11.1 [7.0; 14.1]	8.2 [4.3; 12.0]	9.2 [4.7; 13.5]	9.7 [5.8; 13.3]
Range	1.0–24.0	1.0–23.9	1.0–23.3	1.0–23.9	1.0–24.0
Cumulative follow-up (person-years)	3,881,595	2,800,339	133,542	794,788	7,610,263
Crude incidence (95% CI, per 10,000 person-years)	6.10 (5.86–6.35)	11.84 (11.44–12.25)	4.64 (3.56–5.95)	7.94 (7.33–8.58)	8.38 (8.17–8.59)
Age at the end of follow-up, years					
Mean ± S.D	41.3 ± 11.7	49.9 ± 11.8	37.5 ± 10.6	40.7 ± 12.2	44.1 ± 12.5
Median [IQR]	39.7 [31.6; 49.6]	50.5 [41.7; 58.8]	35.2 [28.8; 44.5]	38.7 [30.3; 49.3]	43.7 [33.5; 53.3]
Range	17.4–73.3	18.3–73.5	18.0–71.3	18.3–73.6	17.4–73.6
Age at obesity-related cancer diagnosis, years					
Mean ± S.D	51.3 ± 10.7	55.2 ± 8.4	50.1 ± 11.0	51.3 ± 10.7	53.3 ± 9.8
Median [IQR]	53.6 [44.3; 59.2]	56.3 [50.8; 61.2]	53.8 [42.6; 57.8]	53.4 [43.3; 59.3]	55.0 [47.9; 60.3]
**Early-onset obesity-related cancers**					
Number of incident cancers	876	747	26	237	1886
Follow-up					
Median [IQR]	8.1 [4.4; 11.4]	7.9 [4.1; 11.4]	7.5 [3.9; 11.0]	7.9 [4.0; 12.0]	8.0 [4.3; 11.4]
Crude incidence (95% CI, per 10,000 person-years)	2.73	4.29	2.16	3.62	3.29
Age at obesity-related cancer diagnosis, years					
Mean ± S.D	38.7 ± 8.7	44.1 ± 7.5	36.0 ± 8.5	38.0 ± 9.0	40.2 ± 8.8
Median [IQR]	38.5 [31.1; 47.5]	47.8 [39.5; 50.0]	34.7 [28.6; 43.3]	37.5 [29.8; 47.2]	41.4 [32.5; 50.0]

The definition of obesity-related cancers was determined according to the International Agency for Research on Cancer (IARC). This encompassed 11 specific cancers: esophagus, postmenopausal breast, liver and gallbladder, stomach, pancreas, colon and rectum, kidney, multiple myeloma, thyroid, uterus, and ovary. Follow-up extended from one year following adult BMI measurements until cancer diagnosis, death, transfer to other state-mandated health provider, or December 16, 2021, whichever came first.

S.D, standard deviation; IQR, interquartile range; HR, hazard ratio; CI, confidence interval.

The above findings persisted when accounting for missing covariate data (Supplementary Table S6) and when the analysis was stratified by sex (Supplementary Table S7), limited to people with unimpaired health at adolescence (Supplementary Table S8), adjusted for smoking and diabetes status (Fig. 2), and excluded those who underwent bariatric surgery (Supplementary Table S9). Including hematological malignancies in the outcome yielded similar results (Supplementary Table S10). Limiting obesity-related cancer diagnosis to age ≤50 years yielded HRs of 1.33 (95% CI, 1.20–1.47), 0.88 (95% CI, 0.60–1.31), and 1.39 (95% CI, 1.20–1.61) for the lean-to-high, high-to-lean, and high-to-high BMI groups, respectively (Fig. 2). Point estimates were similar when limiting diagnoses up to the age of 40 years (Supplementary Table S11). The results persisted across analyses that addressed potential biases related to variation in ages of adult BMI measurement and the interval between BMI assessments (Supplementary Tables S12–S14). Among 598,279 (75%) people with an additional BMI measurement at adulthood, 517,053 (86%) retained their pre-determined trajectories. The corresponding HRs were 1.41 (95% CI, 1.32–1.52), 1.04 (95% CI, 0.72–1.49), and 1.59 (95% CI, 1.43–1.76) for the lean-to-high, high-to-lean, and high-to-high BMI groups, respectively (Supplementary Table S15). The mean adolescent BMI was lower for the high-to-lean than the high-to-high BMI group (27.4 ± 2.7 versus 28.6 ± 3.2 kg/m^2^). However, changes in point estimates were negligible after including adolescent BMI in the model using an adolescent BMI-matched group (Supplementary Table S16). Stratification of high BMI into overweight and obesity showed a graded increase in obesity-related cancer risk. Compared to those who remained lean, individuals who became overweight had an HR of 1.23 (95% CI, 1.16–1.31) and individuals who became obese had an HR of 1.49 (95% CI, 1.39–1.61) (Table 3). HRs for obesity-related cancers were similar for individuals with adolescent obesity who transitioned to overweight (HR 1.10, 95% CI, 0.68–1.77) or to lean BMI (HR 1.16, 95% CI, 0.60–2.23), compared to the lean-to-lean BMI group (Table 3). Results were materially unchanged when the model was repeated with follow-up starting at the adolescent BMI measurement (Supplementary Table S17).

**Table 3:** Stratifying high BMI into overweight and obesity.

	Adulthood BMI		
	Underweight/Healthy weight	Overweight	Obesity
Adolescent BMI			
Underweight/Healthy weight	reference	1.23 (1.16–1.31)	1.49 (1.39–1.61)
Overweight	0.99 (0.75–1.31)	1.33 (1.13–1.57)	1.54 (1.37–1.74)
Obesity	1.16 (0.60–2.23)	1.10 (0.68–1.77)	1.59 (1.32–1.92)

The adjusted model was applied. Hazard ratios and 95% confidence intervals are shown for each trajectory group.

BMI, body mass index.

Individuals were grouped into 9 adolescent-to-adulthood body mass index (BMI) trajectory groups. The three adolescent categories were: Underweight/healthy weight (BMI <85th percentile), overweight (85th–95th percentile) and obesity (≥95th percentile). The three adult categories were: Underweight/healthy weight (BMI <25 kg/m^2^), overweight (BMI of 25–30 kg/m^2^) and obesity (BMI ≥30 kg/m^2^).

### Adolescence-to-adulthood percent weight change and cancer-specific risk

**Fig. 3:**
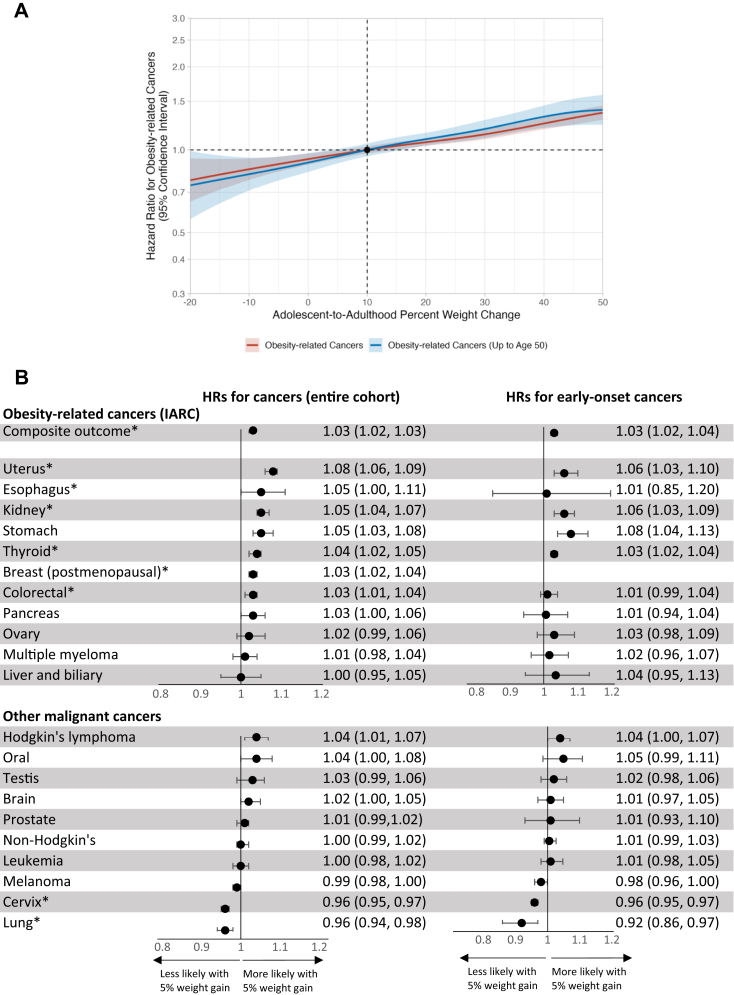
**Adolescent-to-adulthood percent weight change and the risk for specific cancers. A.** Spline models were derived for the hazard ratios (HRs) between percent weight change (calculated as the percentage of adult-adolescence weight difference out of the adolescent weight) and incident obesity-related cancers for the entire cohort as well as for early-onset cancers. The adjusted models were applied, with additional adjustment for adolescent body mass index (BMI). The median percent weight change of the lean-to-lean BMI group was used as a reference (10%), the lines indicate the hazard ratios, and the shading indicates 95% confidence intervals. **B.** Cox proportional models using percent weight change as a continuous variable, per 5 unit increments. The adjusted model was applied. ∗ P-value for linearity <0.05.

**Fig. 4:**
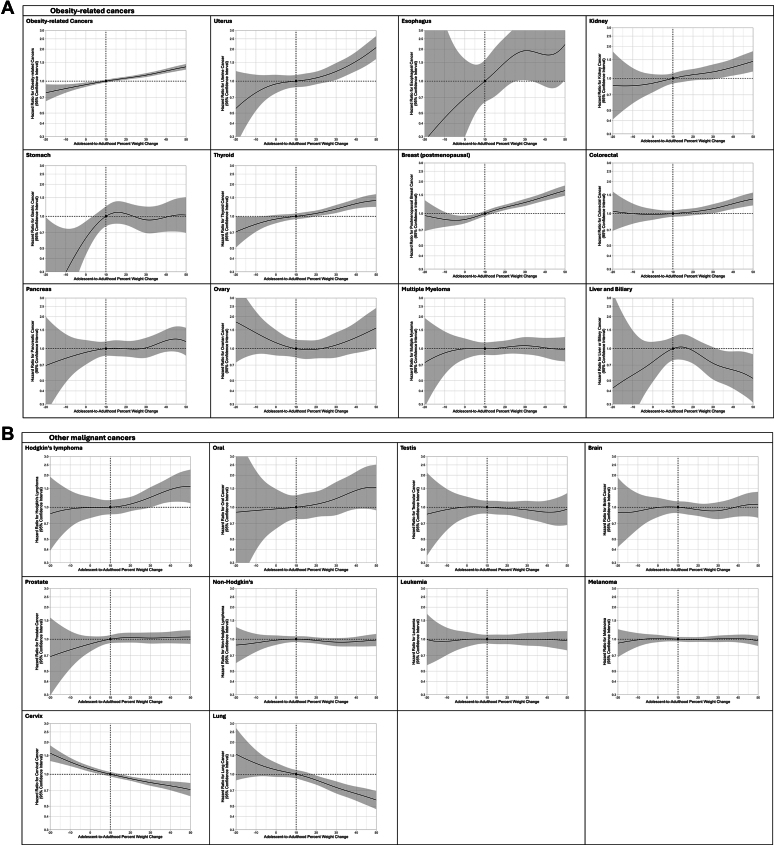
**Spline models for specific cancers:(A)obesity-relatedand(B)other malignant cancers.** Penalized spline models were derived from adjusted Cox proportional hazards models to depict the association between percent weight change from adolescence to adulthood and cancer risk. The adjusted models were applied, with additional adjustment for adolescent BMI. The median percent weight change of the lean-to-lean BMI group (10%) served as the reference point. Solid lines represent hazard ratios, and shaded areas represent 95% confidence intervals.

Cox regression spline models (Fig. 3A, Supplementary Figure S2) show fully adjusted HRs for obesity-related cancers, following additional adjustment for adolescent BMI across a continuum of adolescent-to-adulthood percent weight and delta BMI. The curves for the entire cohort and for early-onset cancers were very similar, indicating consistent risk patterns across both groups (Fig. 3A). Significant linear trends (P < 0.0001) were observed, but not non-linear ones (P = 0.81, P = 0.44). Interactions of adolescent lean/high BMI status with percent weight change (P = 0.95) or delta BMI (P = 0.104) were not found. Splines showing HRs for each cancer type across percent weight change are depicted in Fig. 4. In a linear adjusted Cox model, each 5% weight gain from adolescence, was associated with a 3% (95% CI, 1.02–1.03) increased hazard for obesity-related cancers. This association remained for early-onset cancers, with a 3% increased hazard (95% CI, 1.02–1.04). Specific cancer risks for both the entire cohort and early-onset cancers are shown in Fig. 3B. The specific cancer risks (shown in Fig. 3B) were 3% (95% CI, 1.02–1.04) for post-menopausal breast cancer, 3% (95% CI, 1.01–1.04) for colorectal cancer, 4% (95% CI, 1.02–1.05) for thyroid cancer, 5% (95% CI, 1.04–1.07) for kidney cancer, and 8% for uterine cancer (95% CI, 1.06–1.09). The linear trend was P < 0.001 for each. Some cancers, including leukemia, non-Hodgkin's lymphoma, and malignant brain cancers were not associated with a 5% weight gain but were positively associated with adolescent high BMI (Supplementary Table S18). Linear analysis for each 5-kg weight gain is presented in Supplementary Table S19.

## Discussion

In this cohort of 800,024 people, the risk for all obesity-related cancers was increased by 31% among those with a normal BMI in adolescence and a high BMI in adulthood, and by 47% among those with a high BMI in both periods. Among individuals with a high BMI in adolescence, those with a normal BMI in adulthood had lower cancer risk. Comparable associations were observed when restricting the outcome to early-onset cancers (<50 years). This effect was consistent across different sensitivity analyses. Each 5% weight gain from adolescence to adulthood was associated with a 3% higher hazard for all obesity-related cancers and early-onset cancers, and adolescent obesity was associated with some types of cancer independent of weight trajectory.

High BMI in adulthood is strongly associated with the risk of at least 12 malignant cancers.^6^^,^^30^ Gaining or maintaining a high BMI in adulthood was positively associated with obesity-related cancers, point estimates were reported as 1.10–1.99.^31^^,^^32^ Previous evaluations of weight change from early life relied mostly on recalled weights rather than measured data.^29^^,^^33^^,^^34^ Among 118,140 health professionals, every 5-kg increase in self-reported weight from age 18 to 55 years was associated with an adjusted HR of 1.06 (95% CI, 1.02–1.09) for obesity-related cancers, regardless of initial weight.^29^ Our HR of 1.05 (95% CI, 1.04–1.06) for every 5-kg increment, based on measured data, confirms these findings. Notably, as their follow-up began at age 55 years, the risk for early-onset cancers was not assessed. Data are especially limited for associations with specific cancer types,^30^ including hematologic malignancies.^6^ Nevertheless, our findings corroborate data supporting associations with these malignancies.^27^^,^^35^^,^^36^

The significance of weight status at various life stages for future cancer risk is unclear.^37^ The observation in the current study that normalizing BMI in young adulthood was associated with a lower risk for obesity-related cancers, emphasizes the importance of adulthood BMI. In contrast, the risk for coronary artery disease inferred by adolescent obesity remains elevated regardless of adult BMI, after adjusting for biochemical, lifestyle, and sociodemographic variables.^38^ The independent associations of adolescent BMI and BMI trajectory with most obesity-related cancers suggest that longer durations of high BMI may carry greater risk.^27^ Other cancers may be influenced profoundly by the timing of excess adiposity.^39^ For instance, we observed an inverse relation between high BMI in adolescence and post-menopausal breast cancer risk, alongside a positive association with high BMI during adulthood, as reported elsewhere.^40^, ^41^, ^42^, ^43^ Regarding hematological malignancies, while Hodgkin's lymphoma risk was associated with both adolescent BMI and its trajectory to adulthood, non-Hodgkin's lymphoma and leukemia may be more influenced by adolescent BMI.^36^ These findings highlight the complexity of obesity-related cancer pathogenesis, especially in the early decades of life.

This study has considerable public health implications, given the rising incidences of both obesity and cancer among young adults.^8^^,^^11^ Early-onset cancers are more likely to manifest at advanced stages and result in poorer survival outcomes.^8^ Survivors face elevated risks of long-term complications, including infertility, cardiovascular diseases, and secondary cancers,^44^, ^45^, ^46^ leading to negative social and financial impacts. Notably, 16% of individuals with high BMI in adolescence transitioned to a normal BMI in adulthood (16,248 out of 102,249), though this pattern was observed in only 2% of the entire cohort (16,248 out of 800,024). Our observation that nearly two-thirds of adults with excess weight were lean as adolescents corroborates other reports.^47^ Among 9.6 million adults in the UK, age 18–24 years, followed by 25–34 years, was the most susceptible period to develop overweight or obesity.^48^ This underscores for clinicians and policymakers the importance of early adulthood as a crucial window for weight management in mitigating risks of early-onset cancer.

This study has several limitations. First, due to its population-based design, structured BMI measurements in adulthood were unavailable for 30% of the source population; while for those included, measurements were taken at varying ages. We conducted several sensitivity and subgroup analyses, including limiting adult BMI measurements to certain age groups, matching analysis, and verifying trajectories with a second adult BMI assessment. The extent of weight gain from adolescence to mid-adulthood reported here is comparable to that observed in the general US population.^29^^,^^49^ Further, because up to two adult BMI measurements were available, we could not model full longitudinal BMI trajectories. A second limitation is the unavailability of smoking status during adolescence, and the lack of data on smoking amount and duration. However, adjusting for adult smoking status (never versus ever) did not significantly alter the results. Third, lifestyle habits such as alcohol consumption and physical activity, and family history of diseases were not collected. Importantly, physical activity may reduce cancer risk independent of weight loss.^50^ Fourth, we lacked biochemical data to elucidate underlying mechanisms, such as markers of low-grade inflammation and insulin regulation. Fifth, data on weight-loss causes were unavailable; we could not distinguish weight change due to unintentional weight loss, diet, physical activity, pharmacotherapy, or disease. The positive association between the high-to-lean BMI group and pancreatic cancer may be influenced by unintentional weight loss. To mitigate reverse causality, we excluded those whose BMI was measured less than one year before cancer diagnosis.^14^ In addition, our exclusion at pre-recruitment of individuals with chronic medical treatment, a requirement for ongoing medical follow-up, or a history of major surgery likely reduced some of the disease-related weight change. Nevertheless, heterogeneous weight-change mechanisms remain unmeasured and may have nonequivalent effects on cancer risk, implying a potential violation of the consistency assumption and possible attenuation or obscuring of subtype-specific associations. Sixth, the population was drawn from a single state-mandated health provider; however, the database's nationwide representativeness has been established.^23^ Seventh, remaining underreporting of incident cancer, particularly in outpatient or hematological cases, may differentially affect the BMI trajectory groups. However, given the relatively high case completeness of the registry, this is unlikely to significantly impact the overall findings. Finally, statistical power was lower for a comprehensive analysis of some specific cancer types. Strengths of the study include the integration of two population-based databases with measurements of weight and height, systematic collection of sociodemographic variables, and diverse genetic ancestry.^51^

In conclusion, we report an association between weight trajectories from adolescence to adulthood and the risk of obesity-related cancers, evident as early as age 50 years. Early-onset obesity-related cancers were more common among people who transitioned from a lean status in adolescence to overweight or obesity in adulthood, or who maintained a consistently high BMI. Importantly, normalization of BMI in young adulthood may mitigate this heightened risk. These findings highlight the crucial role of early-life weight management strategies in addressing the burden of early-onset cancers.

## Contributors

C.D.B. and A.B. wrote the original draft. C.D.B., A.B., E.D., and G.T. performed the formal analysis and directly accessed and verified the underlying data reported in the manuscript. C.D.B, A.B., and G.T. conceptualized the study and designed the methodology. A.M.T., I.I.S., L.B., D.D., E.D., B.B., A.T., A.A., R.S.R. and G.C. reviewed and edited the manuscript. All authors read and approved the final version of the manuscript. G.T. supervised the study. C.D.B, A.B. and G.T. are the guarantors of this work and, as such, had full access to all of the data in the study, verified the data and take responsibility for the integrity of the data and the accuracy of the data analysis.

## Data sharing statement

The data are not publicly available due to governmental and ethical restrictions. Interested parties can contact the corresponding author via email.

## Declaration of interests

I.I.S. and L.B. declare that they are employees of Novo Nordisk and hold Novo Nordisk Stocks. D.D. reports research grants, consulting fees, speaker or advisory board honoraria, travel support, and/or advisory board participation from Eli Lilly, Novo Nordisk, Boehringer Ingelheim, AstraZeneca, Graviton, RAFA, and Hoffman–La Roche, as detailed in his ICMJE disclosure form. All other authors declare no competing interests.
