# Supplementary material for: Adolescent to adulthood weight trajectories and the risk of obesity-related cancers, overall and early-onset: a population-based cohort study
Source: eClinicalMedicine. 2026 Jan 5;91:103711. doi: 10.1016/j.eclinm.2025.103711 (PMC12809738; doi:10.1016/j.eclinm.2025.103711)
Supplement: Supplementary appendix [file mmc1.docx]

**Supplementary Appendix**

**Adolescent to adulthood weight trajectory and early-onset cancer**

| **Cohort build-up** | | |
| --- | --- | --- |
| Supplementary Table S1 | Characteristics at late adolescence of people with vs. without adult BMI measurement | 2-3 |
| Supplementary Figure S1 | Distribution of obesity-related cancers among people with and without adult BMI measurement | 4 |
| Supplementary Table S2 | Comparison of hazards for incident obesity-related cancers between people with and without adult BMI measurements | 5 |
| **BMI trajectory categories and obesity-related cancers** | | |
| Supplementary Table S3 | Number of missing values for baseline characteristics of study cohort | 6 |
| Supplementary Table S4 | Characteristics of people diagnosed with obesity-related cancers. | 7 |
| Supplementary Table S5 | Incident numbers and ages at cancer diagnosis | 8 |
| Supplementary Table S6 | Assessment of potential bias due to missing data | 9 |
| Supplementary Table S7 | Analysis stratified by men and women | 10 |
| Supplementary Table S8 | A sensitivity analysis limited to people with unimpaired health at adolescence | 11 |
| Supplementary Table S9 | A sensitivity analysis that excluded people who underwent bariatric surgery | 12 |
| Supplementary Table S10 | Adding hematological malignancies to the outcome of obesity-related cancers | 13 |
| Supplementary Table S11 | Setting the outcome as obesity-related cancers diagnosed up to the age of 50 and 40 years. | 14 |
| Supplementary Table S12 | Sensitivity analyses controlling for cohort differences in the timing of adult BMI measurement by median age, birth period, and measurement era | 15-16 |
| Supplementary Table S13 | Sensitivity analysis stratified by ages at adult BMI measurement | 17 |
| Supplementary Table S14 | Matched cohort analysis controlling for both age at adult BMI measurement and interval between adolescent and adult BMI measurements | 18-19 |
| Supplementary Table S15 | Defining BMI trajectories based on two time points in adulthood | 20 |
| Supplementary Table S16 | Stringently controlling for baseline BMI | 21 |
| Supplementary Table S17 | Sensitivity analysis with follow-up starting at the adolescent BMI measurement | 22 |
| **Linear analyses of weight change and cancer-specific risk** | | |
| Supplementary Figure S2 | A spline model across adulthood-adolescent BMI difference | 23 |
| Supplementary Table S18 | A linear adjusted model using adolescent BMI and adolescent-to-adulthood delta BMI as continuous variables | 24-26 |
| Supplementary Table S19 | A linear adjusted model of each 5-kg weight gain from body weight at adolescence | 27 |

**Supplementary Table S1: Characteristics at late adolescence of people with vs. without adult BMI measurement**

|  | **With an adult BMI measurement  (study cohort)** | **Without an adult BMI measurement** |
| --- | --- | --- |
| Number of people | 800,024 | 344,891* |
| Men, number (%) | 438,887 (54.9) | 210,926 (61.2) |
| Women, number (%) | 361,137 (45.1) | 133,965 (38.8) |
| **At late adolescence** | | |
| Age (years) at the first measurement of BMI (pre-recruitment) | 17.3 ± 0.4 | 17.2 ± 0.5 |
| Year of first BMI measurement |  |  |
| Mean ± SD | 1994 ± 12.5 | 2001 ± 15.7 |
| Median [IQR] | 1994 [1985; 2004] | 2004 [1988; 2016] |
| Range | 1967-2019 | 1967-2019 |
| Socioeconomic position, % |  |  |
| Low | 18 | 21 |
| Medium | 52 | 49 |
| High | 30 | 29 |
| Intelligence score, % |  |  |
| Low | 11 | 13 |
| Medium | 72 | 70 |
| High | 17 | 17 |
| Graduated high school, % | 91 | 90 |
| Born in Israel, % | 85 | 82 |
| Unimpaired health,** % | 72 | 72 |
| **At adulthood** | | |
| Diagnosed with diabetes before cancer, number (%) | 46,642 (5.8) | 766 (0.2) |
| Smoking, number (%) | 318,945 (40.2) | 37,437 (18.1) |
| Follow-up, years |  |  |
| Mean ± SD | 9.5 ± 4.5 | NA |
| **At the end of follow-up**† | | |
| Age at the end of follow-up |  |  |
| Mean ± S.D | 44.1 ± 12.5 | 27.7 ± 9.2 |
| Median [IQR] | 43.7 [33.5; 53.3] | 24.5 [20.9; 32.3] |
| Range | 17.4 – 73.6 | 16.7 – 71.7 |
| Follow-up (from adolescent BMI measurement), years |  |  |
| Mean ± SD | 26.7 ± 12.4 | 10.4 ± 9.1 |
| Median [IQR] | 26.3 [16.3;35.9] | 7.3 [3.6;14.9] |
| Range | 2.6 – 55.0 | 1.0 – 55.0 |

The variables were defined as described in Table 1.

* Among 360,512 people without data of an adult BMI measurement, 15,621 had data of less than one year follow-up, and were thus excluded from the analysis.

** Unimpaired health was defined for people without any morbidities at their adolescent medical evaluation that required medical follow-up or chronic treatment and without a history of cancer or major surgery.

† For this comparison, follow-up (both in the study cohort and in the cohort without adult BMI measurement) was calculated from the adolescent BMI measurement until cancer diagnosis, death, transfer to another state-mandated health provider, or December 16, 2021, whichever came first.

People without adult BMI measurements exhibited comparable sociodemographic characteristics to those with adult BMI measurement (study cohort). Nevertheless, their adolescent evaluation occurred more recently (the median year was 2004 compared to 1994), resulting in a younger mean age at the end of follow-up (27.7 ± 9.2 versus 44.1 ± 12.5 years, P<0.0001) and shorter mean follow-up (10.4 ± 9.1 versus 26.7 ± 12.4).

BMI, body mass index; SD, standard deviation; IQR, interquartile range

**Supplementary Figure S1: Distribution of obesity-related cancers among people with and without adult BMI measurement**

1. In our cohort of 800,024 people, 6,376 had obesity-related cancers: 1,836 post-menopausal breast, 1,208 colorectal, 48 esophagus, 521 kidney, 110 liver and biliary, 346 multiple myeloma, 122 ovary, 277 pancreas, 183 stomach, 1,397 thyroid, and 340 uterus.
2. Among people without adult BMI measurement (n=344,891), 711 had obesity-related cancers: 37 post-menopausal breast, 192 colorectal, 11 esophagus, 20 kidney, 13 liver and biliary, 29 multiple myeloma, 53 ovary, 77 pancreas, 32 stomach, 219 thyroid, and 29 uterus.

As the mean age of the people without adult BMI data was younger at the end of the follow-up than that of the study cohort (those with adult BMI measurements), the distribution of incident cancers differed, manifesting a lower fraction of cancers typically associated with older ages, such as postmenopausal breast cancers.

**Supplementary Table S2: Comparison of hazards for incident obesity-related cancers between people with and without adult BMI measurements**

|  | **Adolescent lean BMI** | **Adolescent high BMI** | **Total** |
| --- | --- | --- | --- |
| **With adult BMI measurement (n = 800,024)*** | | | |
| Number of people | 683,508 | 100,512 | 784,020 |
| Diagnosed with obesity-related cancers | 5,311 | 661 | 5,972 |
| HR | 1 (reference) | 1.32 |  |
| 95% CI |  | 1.22 – 1.43 |  |
| P value |  | <0.001 |  |
| **Without adult BMI measurement^*^** | | | |
| Number of people | 286,141 | 47,274 | 333,415 |
| Diagnosed with obesity-related cancers | 592 | 75 | 667 |
| HR | 1 (reference) | 1.42 |  |
| 95% CI |  | 1.12 – 1.81 |  |
| P value |  | 0.004 |  |

*In these analyses, the follow-up (both for people with and without an adult BMI measurement) was calculated from the adolescent BMI measurement until cancer diagnosis, death, transfer to another state-mandated health provider, or December 16, 2021, whichever came first. This contrasts to the primary analysis in which the follow-up extended for one year following an adult BMI measurement. Note that compared to adolescent lean BMI, high BMI at adolescence was associated with an increased risk for obesity-related cancers, with similar point estimates to the main analysis.

BMI, body mass index; HR, hazard ratio; CI, confidence interval

**Supplementary Table S3: Number of missing values for baseline characteristics of study cohort**

|  | **Lean-to-Lean BMI** | **Lean-to-High BMI** | **High-to-Lean BMI** | **High-to-High BMI** | **Total** |
| --- | --- | --- | --- | --- | --- |
| Residential socioeconomic position, % | 4,867 (1.1) | 4,834 (1.8) | 118 (0.7) | 855 (1.0) | 10,674 (1.3) |
| Intelligence score, % | 3,236 (0.8) | 3,398 (1.3) | 123 (0.8) | 813 (0.9) | 7.570 (0.9) |
| Graduated high school, % | 1,403 (0.3) | 1,478 (0.5) | 43 (0.3) | 269 (0.3) | 3,193 (0.4) |
| Born in Israel, % | 129 (0.0) | 175 (0.1) | 4 (0.0) | 24 (0.0) | 332 (0.0) |
| Country of origin | 2,778 (0.6) | 2,349 (0.9) | 106 (0.7) | 671 (0.8) | 5,904 (0.7) |
| Smoking, number (%) | 3,037 (0.7) | 2,142 (0.8) | 177 (1.1) | 942 (1.1) | 6,298 (0.8) |

**Supplementary Table S4: Characteristics of people diagnosed with obesity-related cancers**

|  | **Diagnosed with obesity-related cancers** | **Not diagnosed with obesity-related cancers** |
| --- | --- | --- |
| People | 6,376 | 793,648 |
| Men, number (%) | 2,205 (34.6) | 436,682 (55.0) |
| Women, number (%) | 4,171 (65.4) | 356,966 (45.0) |
| BMI at late adolescence, kg/m^2^ | 21.7 ± 3.1 | 21.7 ± 3.4 |
| BMI at adulthood, kg/m^2^ | 27.2 ± 5.5 | 25.1 ± 4.8 |
| Delta BMI, kg/m^2^ | 5.5 ± 4.7 | 3.4 ± 4.0 |
| Delta weight, kg | 17.1 ± 14.0 | 11.6 ± 12.6 |

The definition of obesity-related cancers was determined according to the International Agency for Research on Cancer^[[1]](#footnote-2)^, encompassing eleven malignant cancers: esophagus, postmenopausal breast, liver and gallbladder, stomach, pancreas, colon and rectum, kidney, multiple myeloma, thyroid, uterus, and ovary.

BMI, body mass index

**Supplementary Table S5: Incident numbers and ages at cancer diagnosis**

| **Type of cancers** | **Number of people** | **Age at cancer diagnosis** |
| --- | --- | --- |
| 1. Obesity-related cancers (IARC) | |  |
| All obesity-related cancers (IARC) | 6,376* | 53.3 ± 9.8 |
| Breast (post-menopausal) | 1,836 | 58.8 ± 4.6 |
| Colorectal | 1,208 | 53.5 ± 9.0 |
| Esophagus | 48 | 57.8 ± 7.4 |
| Kidney | 521 | 52.6 ± 9.1 |
| Liver and biliary | 110 | 55.8 ± 8.7 |
| Multiple myeloma | 346 | 56.2 ± 8.4 |
| Ovary | 122 | 50.4 ± 9.2 |
| Pancreas | 277 | 56.1 ± 7.9 |
| Stomach | 183 | 53.0 ± 9.3 |
| Thyroid | 1,397 | 44.3 ± 10.2 |
| Uterus | 340 | 55.2 ± 8.6 |
| 1. Other malignant cancers | |  |
| Brain | 343 | 48.3 ± 11.6 |
| Cervix | 2,905 | 36.6 ± 9.0 |
| Hodgkin's lymphoma | 224 | 39.1 ± 11.1 |
| Leukemia | 596 | 52.8 ±10.8 |
| Lung | 697 | 56.8 ± 7.7 |
| Melanoma | 2,227 | 49.7 ± 10.7 |
| Non-Hodgkin's lymphoma | 1,426 | 50.5 ± 10.7 |
| Oral | 99 | 50.2 ± 10.7 |
| Prostate | 1,337 | 60.7 ± 5.6 |
| Testis | 285 | 37.9 ± 8.6 |

*Of 6,376 people with cancer, 10 were diagnosed with two types of obesity-related cancers on the same date, and one was diagnosed with three types of obesity-related cancers.

**Supplementary Table S6: Assessment of potential bias due to missing data**

**Part A**

|  | **HR (95% CI)** | **P value** |
| --- | --- | --- |
| Weight status |  |  |
| Lean-to-High BMI | 1.32 (1.25 – 1.39) | <0.001 |
| High-to-Lean BMI | 1.00 (0.77 – 1.28) | 0.970 |
| High-to High BMI | 1.46 (1.34 – 1.60) | <0.001 |
| Sex | 2.95 (2.80 – 3.11) | <0.001 |
| Year at adulthood measurement | 0.93 (0.92 – 0.94) | <0.001 |
| Age at adulthood measurement | 1.10 (1.10 – 1.11) | <0.001 |
| Missing data | 1.06 (0.96 – 1.18) | 0.241 |

Hazard ratios (HRs) and 95% confidence intervals (CIs) for obesity-related cancers according to BMI trajectory groups, adjusted for sex, age at adult BMI measurement, and year of adult BMI measurement, with an additional binary variable indicating the presence of any missing covariate data. The “missing data” term was not statistically significant (P = 0.241).

**Part B**

|  | **Lean-to-Lean BMI** | **Lean-to-High BMI** | **High-to-Lean BMI** | **High-to-High BMI** | **Total** |
| --- | --- | --- | --- | --- | --- |
| **Minimally adjusted** |  |  |  |  |  |
| Number of people | 429,038 | 268,737 | 16,283 | 85,966 | 800,024 |
| Diagnosed with obesity-related cancers | 2,368 | 3,315 | 62 | 631 | 6,376 |
| HR | 1 (reference) | 1.31 | 1.01 | 1.47 |  |
| 95% CI |  | 1.24 – 1.39 | 0.78 – 1.31 | 1.34 – 1.61 |  |
| P value |  | <0.001 | 0.919 | <0.001 |  |
| **Those without missing data** |  |  |  |  |  |
| Number of people | 421,865 | 261,643 | 16,065 | 84,447 | 784,020 |
| Diagnosed with obesity-related cancers | 2,235 | 3,076 | 60 | 601 | 5,972 |
| HR | 1 (reference) | 1.31 | 1.01 | 1.47 |  |
| 95% CI |  | 1.24 – 1.39 | 0.78 – 1.31 | 1.34 – 1.61 |  |
| P value |  | <0.001 | 0.928 | <0.001 |  |
| **Those with missing data** |  |  |  |  |  |
| Number of people | 7,173 | 7,094 | 218 | 1,519 | 16,004 |
| Diagnosed with obesity-related cancers | 133 | 239 | 2 | 30 | 404 |
| HR | 1 (reference) | 1.43 | 0.66 | 1.34 |  |
| 95% CI |  | 1.15 – 1.77 | 0.16 – 2.67 | 0.89 – 1.99 |  |
| P value |  | 0.001 | 0.561 | 0.158 |  |

Hazard ratios (HRs) and 95% confidence intervals (CIs) for obesity-related cancers by BMI trajectory groups, presented separately for participants with and without missing covariate data. Estimates in the subgroup with missing data are less precise owing to the small sample size, but the direction of associations was broadly consistent with the main findings.

**Supplementary Table S7: Analysis stratified by men and women**

|  | **Lean-to-Lean BMI** | **Lean-to-High BMI** | **High-to-Lean BMI** | **High-to-High BMI** | **Total** |
| --- | --- | --- | --- | --- | --- |
| **Main analysis** | | | | | |
| Number of people | 421,865 | 261,643 | 16,065 | 84,447 | 784,020 |
| Diagnosed with obesity-related cancers | 2,235 | 3,076 | 60 | 601 | 5,972 |
| HR | 1 (reference) | 1.31 | 1.01 | 1.47 |  |
| 95% CI |  | 1.24 – 1.39 | 0.78 – 1.31 | 1.34 – 1.61 |  |
| P value |  | <0.001 | 0.919 | <0.001 |  |
| **Men** | | | | | |
| Number of people | 195,076 | 178,716 | 7,345 | 50,607 | 431,744 |
| Diagnosed with obesity-related cancers | 517 | 1,359 | 10 | 257 | 2,143 |
| HR | 1 (reference) | 1.32 | 1.01 | 1.60 |  |
| 95% CI |  | 1.19 – 1.47 | 0.54 – 1.89 | 1.37 – 1.86 |  |
| P value |  | <0.001 | 0.968 | <0.001 |  |
| **Women** | | | | | |
| Number of people | 226,789 | 82,927 | 8,720 | 33,840 | 352,276 |
| Diagnosed with obesity-related cancers | 1,718 | 1,717 | 50 | 344 | 3,829 |
| HR | 1 (reference) | 1.32 | 1.03 | 1.41 |  |
| 95% CI |  | 1.23 – 1.42 | 0.77 – 1.36 | 1.25 – 1.58 |  |
| P value |  | <0.001 | 0.855 | <0.001 |  |

The main analysis is shown to facilitate comparison. The model (main analysis) was adjusted for sex, age at the adult BMI measurement, the year of the adult BMI measurement, education, intelligence score, residential socioeconomic status, and country of birth.

BMI, body mass index; HR, hazard ratio; CI, confidence interval

|  | **Lean-to-Lean BMI** | **Lean-to-High BMI** | **High-to-Lean BMI** | **High-to-High BMI** | **Total** |
| --- | --- | --- | --- | --- | --- |
| **Main analysis** | | | | | |
| Number of people | 421,865 | 261,643 | 16,065 | 84,447 | 784,020 |
| Diagnosed with obesity-related cancers | 2,235 | 3,076 | 60 | 601 | 5,972 |
| HR | 1 (reference) | 1.31 | 1.01 | 1.47 |  |
| 95% CI |  | 1.24 – 1.39 | 0.78 – 1.31 | 1.34 – 1.61 |  |
| P value |  | <0.001 | 0.919 | <0.001 |  |
| **People with unimpaired health at adolescence** | | | | | |
| Number of people | 350,116 | 224,646 | 12,715 | 66,857 | 654,334 |
| Diagnosed with obesity-related cancers | 2,010 | 2,813 | 57 | 508 | 5,388 |
| HR | 1 (reference) | 1.30 | 1.10 | 1.43 |  |
| 95% CI |  | 1.23 – 1.39 | 0.84 – 1.43 | 1.29 – 1.58 |  |
| P value |  | <0.001 | 0.480 | <0.001 |  |

**Supplementary Table S8: A sensitivity analysis limited to people with unimpaired health*** **at adolescence**

*Unimpaired health was as the absence of morbidities at the adolescent medical evaluation that required medical follow-up or chronic treatment and without a history of cancer or major surgery. The main analysis is shown to facilitate comparison. The adjusted model was applied.

BMI, body mass index; HR, hazard ratio; CI, confidence interval

**Supplementary Table S9: A sensitivity analysis that excluded people who underwent bariatric surgery**

|  | **Lean-to-Lean BMI** | **Lean-to-High BMI** | **High-to-Lean BMI** | **High-to-High BMI** | **Total** |
| --- | --- | --- | --- | --- | --- |
| **Main analysis** | | | | | |
| Number of people | 421,865 | 261,643 | 16,065 | 84,447 | 784,020 |
| Diagnosed with obesity-related cancers | 2,235 | 3,076 | 60 | 601 | 5,972 |
| HR | 1 (reference) | 1.31 | 1.01 | 1.47 |  |
| 95% CI |  | 1.24 – 1.39 | 0.78 – 1.31 | 1.34 – 1.61 |  |
| P value |  | <0.001 | 0.919 | <0.001 |  |
| **Excluding people who underwent bariatric surgery** | | | | | |
| Number of people | 421,267 | 256,421 | 15,834 | 76,661 | 770,183 |
| Diagnosed with obesity-related cancers | 2,213 | 2,969 | 58 | 520 | 5,760 |
| HR | 1 (reference) | 1.33 | 1.00 | 1.50 |  |
| 95% CI |  | 1.25 – 1.40 | 0.77 – 1.30 | 1.36 – 1.65 |  |
| P value |  | <0.001 | 0.981 | <0.001 |  |

As bariatric surgery may decrease obesity-related cancer incidence, people who underwent bariatric surgery were excluded from this analysis. The people who underwent bariatric surgeries were identified from the Maccabi Healthcare Service dataset. The main analysis is shown to facilitate comparison. The adjusted model was applied.

HR, hazard ratio; CI, confidence interval

**Supplementary Table S10: Adding hematological malignancies to the outcome of obesity-related cancers**

|  | **Lean-to-Lean BMI** | **Lean-to-High BMI** | **High-to-Lean BMI** | **High-to-High BMI** | **Total** |
| --- | --- | --- | --- | --- | --- |
| **Main analysis** | | | | | |
| Number of people | 421,865 | 261,643 | 16,065 | 84,447 | 784,020 |
| Diagnosed with obesity-related cancers | 2,235 | 3,076 | 60 | 601 | 5,972 |
| HR | 1 (reference) | 1.31 | 1.01 | 1.47 |  |
| 95% CI |  | 1.24 – 1.39 | 0.78 – 1.31 | 1.34 – 1.61 |  |
| P value |  | <0.001 | 0.919 | <0.001 |  |
| **Adding hematological malignancies to the outcome** | | | | | |
| Number of people | 421,865 | 261,643 | 16,065 | 84,447 | 784,020 |
| Diagnosed with obesity-related cancers | 3,091 | 4,102 | 81 | 848 | 8,122 |
| HR | 1 (reference) | 1.23 | 0.99 | 1.43 |  |
| 95% CI |  | 1.17 – 1.29 | 0.79 – 1.23 | 1.32 – 1.54 |  |
| P value |  | <0.001 | 0.895 | <0.001 |  |

Acknowledging the growing literature that suggests a potential association between high BMI and other forms of cancers, we included Hodgkin's lymphoma, non-Hodgkin's lymphoma, and leukemia in the outcome. This analysis yielded similar results. The adjusted model was applied.

BMI, body mass index; HR, hazard ratio; CI, confidence interval

**Supplementary Table S11: Setting the outcome as obesity-related cancers diagnosed up to the age of 50 and 40 years.**

|  | **Lean-to-Lean BMI** | **Lean-to-High BMI** | **High-to-Lean BMI** | **High-to-High BMI** | **Total** |
| --- | --- | --- | --- | --- | --- |
| **Main analysis** | | | | | |
| Number of participants | 421,865 | 261,643 | 16,065 | 84,447 | 784,020 |
| Diagnosed with obesity-related cancers | 2,235 | 3,076 | 60 | 601 | 5,972 |
| HR | 1 (reference) | 1.31 | 1.01 | 1.47 |  |
| 95% CI |  | 1.24 – 1.39 | 0.78 – 1.31 | 1.34 – 1.61 |  |
| P value |  | <0.001 | 0.919 | <0.001 |  |
| **Obesity-related cancers before the age of 50 years** | | | | | |
| Number of participants | 421,865 | 261,643 | 16,065 | 84,447 | 784,020 |
| Diagnosed with obesity-related cancers | 868 | 729 | 26 | 231 | 1,854 |
| HR | 1 (reference) | 1.33 | 0.88 | 1.39 |  |
| 95% CI |  | 1.20 – 1.47 | 0.60 – 1.31 | 1.20 – 1.61 |  |
| P value |  | <0.001 | 0.535 | <0.001 |  |
| **Obesity-related cancers before the age of 40 years** | | | | | |
| Number of participants | 421,865 | 261,643 | 16,065 | 84,447 | 784,020 |
| Diagnosed with obesity-related cancers | 426 | 203 | 13 | 110 | 752 |
| HR | 1 (reference) | 1.25 | 0.78 | 1.33 |  |
| 95% CI |  | 1.05 – 1.49 | 0.45 – 1.35 | 1.08 – 1.65 |  |
| P value |  | 0.012 | 0.376 | 0.008 |  |

The association persisted in early-onset cancers (up to the age of 50 years), and in cancers diagnosed during young adulthood (up to the age of 40 years). The main analysis is shown to facilitate comparison. The adjusted model was applied for all analyses.

HR, hazard ratio; CI, confidence interval

**Supplementary Table S12: Sensitivity analyses controlling for cohort differences in the timing of adult BMI measurement by median age, birth period, and measurement era**

|  | **Lean-to-Lean BMI** | **Lean-to-High BMI** | **High-to-Lean BMI** | **High-to-High BMI** | **Total** |
| --- | --- | --- | --- | --- | --- |
| **Main analysis** | | | | | |
| Number of people | 421,865 | 261,643 | 16,065 | 84,447 | 784,020 |
| Diagnosed with obesity-related cancers | 2,235 | 3,076 | 60 | 601 | 5,972 |
| HR | 1 (reference) | 1.31 | 1.01 | 1.47 |  |
| 95% CI |  | 1.24 – 1.39 | 0.78 – 1.31 | 1.34 – 1.61 |  |
| P value |  | <0.001 | 0.919 | <0.001 |  |
| **The age at the adult measurement was lower or equal to the median^*^** | | | | | |
| Number of people | 252,778 | 77,378 | 11,754 | 53,945 | 395,855 |
| Diagnosed with obesity-related cancers | 414 | 201 | 12 | 119 | 746 |
| HR | 1 (reference) | 1.27 | 0.74 | 1.39 |  |
| 95% CI |  | 1.07 – 1.52 | 0.42 – 1.32 | 1.13 – 1.70 |  |
| P value |  | 0.007 | 0.305 | 0.002 |  |
| **The age at the adult measurement was greater than the median^*^** | | | | | |
| Number of people | 169,087 | 184,265 | 4,311 | 30,502 | 388,165 |
| Diagnosed with obesity-related cancers | 1,821 | 2,875 | 48 | 482 | 5,226 |
| HR | 1 (reference) | 1.33 | 1.10 | 1.49 |  |
| 95% CI |  | 1.25 – 1.41 | 0.83 – 1.47 | 1.35 – 1.65 |  |
| P value |  | <0.001 | 0.496 | <0.001 |  |
| **Year of birth 1980 or before** | | | | | |
| Number of people | 204,372 | 205,170 | 5,454 | 39,071 |  |
| Diagnosed with obesity-related cancers | 2,003 | 2,993 | 54 | 547 |  |
| HR | 1 (reference) | 1.31 | 1.09 | 1.48 |  |
| 95% CI |  | 1.24 – 1.39 | 0.83 – 1.42 | 1.35 – 1.63 |  |
| P value |  | <0.001 | 0.554 | <0.001 |  |
| **Year of birth after 1980** | | | | | |
| Number of people | 217,493 | 56,473 | 10,611 | 45,376 |  |
| Diagnosed with obesity-related cancers | 232 | 83 | 6 | 54 |  |
| HR | 1 (reference) | 1.57 | 0.62 | 1.37 |  |
| 95% CI |  | 1.22 – 2.03 | 0.27 – 1.39 | 1.02 – 1.84 |  |
| P value |  | 0.001 | 0.241 | 0.039 |  |
| **The adolescent evaluation was during or after 1994**^**^ | | | | | |
| Number of people | 263,781 | 82,507 | 12,115 | 53,733 | 412,136 |
| Diagnosed with obesity-related cancers | 367 | 156 | 12 | 89 | 624 |
| HR | 1 (reference) | 1.37 | 0.83 | 1.36 |  |
| 95% CI |  | 1.13 – 1.65 | 0.46 – 1.47 | 1.07 – 1.71 |  |
| P value |  | 0.001 | 0.513 | 0.010 |  |

Three sensitivity analyses were conducted to account for inter-group differences in BMI measurement in adulthood.

*The median age of the adult BMI measurement was 33.1 years.

**Digital documentation of BMI measurements was established at Maccabi Health Services in 1998. For the vast majority of recruits, the maximal period from pre-recruitment medical evaluation until release from mandatory military service is three to four years. Thus, an analysis limited to people who underwent a medical evaluation from 1994 onwards minimized the loss of BMI data.

The main analysis is shown to facilitate comparison. The adjusted model was applied.

BMI, body mass index; HR, hazard ratio; CI, confidence interval.

**Supplementary Table S13: Sensitivity analysis stratified by ages at adult BMI measurement**

|  | **Lean-to-Lean BMI** | **Lean-to-High BMI** | **High-to-Lean BMI** | **High-to-High BMI** | **Total** |
| --- | --- | --- | --- | --- | --- |
| **Main analysis** | | | | | |
| Number of people | 421,865 | 261,643 | 16,065 | 84,447 | 784,020 |
| Diagnosed with obesity-related cancers | 2,235 | 3,076 | 60 | 601 | 5,972 |
| HR | 1 (reference) | 1.31 | 1.01 | 1.47 |  |
| 95% CI |  | 1.24 – 1.39 | 0.78 – 1.31 | 1.34 – 1.61 |  |
| P value |  | <0.001 | 0.919 | <0.001 |  |
| **Age at adult measurement of BMI <30** | | | | | |
| Number of people | 214,663 | 54,463 | 10,500 | 46,087 | 325,713 |
| Diagnosed with obesity-related cancers | 308 | 106 | 7 | 81 | 502 |
| HR | 1 (reference) | 1.17 | 0.55 | 1.30 |  |
| 95% CI |  | 0.93 – 1.47 | 0.26 – 1.16 | 1.02 – 1.67 |  |
| P value |  | 0.172 | 0.114 | 0.038 |  |
| **Age at adult measurement of BMI 30-39** | | | | | |
| Number of people | 114,584 | 84,464 | 3,517 | 22,288 | 224,853 |
| Diagnosed with obesity-related cancers | 424 | 474 | 16 | 128 | 1,042 |
| HR | 1 (reference) | 1.41 | 1.21 | 1.41 |  |
| 95% CI |  | 1.23 – 1.62 | 0.73 – 1.99 | 1.15 – 1.72 |  |
| P value |  | <0.001 | 0.464 | <0.001 |  |
| **Age at adult measurement of BMI 40-49** | | | | | |
| Number of people | 65,906 | 77,105 | 1,555 | 10,799 | 155,365 |
| Diagnosed with obesity-related cancers | 788 | 1,207 | 19 | 212 | 2,226 |
| HR | 1 (reference) | 1.38 | 0.98 | 1.63 |  |
| 95% CI |  | 1.25 – 1.51 | 0.62 – 1.54 | 1.40 – 1.91 |  |
| P value |  | <0.001 | 0.919 | <0.001 |  |
| **Age at adult measurement of BMI >50** | | | | | |
| Number of people | 26,712 | 45,611 | 493 | 5,273 | 78,089 |
| Diagnosed with obesity-related cancers | 715 | 1,289 | 18 | 180 | 2,202 |
| HR | 1 (reference) | 1.25 | 1.33 | 1.43 |  |
| 95% CI |  | 1.13 – 1.37 | 0.83 – 2.12 | 1.21 – 1.69 |  |
| P value |  | <0.001 | 0.232 | <0.001 |  |

Hazard ratios (HRs) and 95% confidence intervals (CIs) for obesity-related cancers according to BMI trajectory groups within each age stratum.

The main analysis is shown to facilitate comparison. The adjusted model was applied.

BMI, body mass index; HR, hazard ratio; CI, confidence interval.

**Supplementary Table S14: Matched cohort analysis controlling for both age at adult BMI measurement and interval between adolescent and adult BMI measurements**

**Part A: Characteristics of adolescent-adulthood BMI trajectory groups**

|  | **Matched Lean-to-Lean BMI** | **Matched Lean-to-High BMI** | **High-to-Lean BMI** | **Matched High-to-High BMI** | **Total** |
| --- | --- | --- | --- | --- | --- |
| Number of people (%) | 48,195 | 48,195 | 16,065 | 48,195 | 160,650 |
| Men, number (%) | 22,035 (45.7) | 22,035 (45.7) | 7,345 (45.7) | 22,035 (45.7) | 73,450 (45.7) |
| Women, number (%) | 26,160 (54.3) | 26,160 (54.3) | 8,720 (54.3) | 26,160 (54.3) | 87,200 (54.3) |
| **At late adolescence** | | | | | |
| Age at evaluation, years Mean ± S.D | 17.3 ± 0.4 | 17.3 ± 0.4 | 17.2 ± 0.4 | 17.3 ± 0.4 | 17.3 ± 0.4 |
| BMI, kg/m2 |  |  |  |  |  |
| Mean ± S.D | 20.2 ± 2.0 | 22.4 ± 1.9 | 27.4 ± 2.7 | 28.8 ± 3.3 | 24.1 ± 4.4 |
| Median [IQR] | 20.1 [18.8;21.6] | 22.6 [21.1;23.8] | 26.6 [25.8;28.1] | 27.8 [26.3;30.1] | 23.7 [20.8;26.7] |
| Residential socioeconomic position, % |  |  |  |  |  |
| Low | 17 | 18 | 17 | 20 | 18 |
| Medium | 52 | 53 | 53 | 55 | 53 |
| High | 31 | 29 | 30 | 25 | 29 |
| Intelligence score, % |  |  |  |  |  |
| Low | 11 | 13 | 13 | 17 | 14 |
| Medium | 73 | 72 | 74 | 71 | 72 |
| High | 16 | 15 | 13 | 12 | 14 |
| Graduated high school, % | 96 | 93 | 95 | 94 | 94 |
| Born in Israel, % | 82 | 80 | 84 | 82 | 82 |
| Country of origin* |  |  |  |  |  |
| Israel | 5,603 (12) | 3,514 (7) | 2,069 (13) | 5,906 (12) | 17,092 (11) |
| Former USSR | 9,639 (20) | 9,675 (20) | 3,031 (19) | 9,885 (21) | 32,230 (20) |
| Asia | 10,079 (21) | 10,330 (21) | 3,059 (19) | 8,856 (19) | 32,324 (20) |
| Africa | 8,309 (17) | 9,486 (20) | 3,123 (20) | 9,281 (19) | 30,199 (19) |
| Europe and north America | 14,219 (30) | 14,917 (31) | 4,671 (29) | 13,906 (29) | 47,713 (30) |
| Ethiopia | 175 (<1) | 95 (<1) | 40 (<1) | 108 (<1) | 418 (<1) |
| Unimpaired health*, number (%) | 39,629 (82) | 40,133 (83) | 12,715 (79) | 37,902 (79) | 130,379 (81) |
| **At adulthood** | | | | | |
| Age at adult measurement of BMI |  |  |  |  |  |
| Mean ± S.D | 29.4 ± 8.5 | 29.7 ± 8.5 | 29.2 ± 8.5 | 29.2 ± 8.5 | 29.4 ± 8.5 |
| Median [IQR] | 26.3 [22.8;34.1] | 26.5 [23.1;34.6] | 25.8 [22.6;33.8] | 26.0 [22.4;33.9] | 26.2 [22.7;34.2] |
| Adult BMI, kg/m2 |  |  |  |  |  |
| Mean ± S.D | 21.6 ± 2.1 | 28.4 ± 3.4 | 23.1 ± 1.6 | 31.6 ± 5.0 | 26.8 ± 5.5 |
| Median [IQR] | 21.8 [20.1;23.3] | 27.4 [26.0;29.9] | 23.5 [22.3;24.3] | 30.5 [27.8;34.4] | 26.0 [23.0;29.7] |
| Delta weight |  |  |  |  |  |
| Kg, Mean ± S.D | 5.5 ± 7.6 | 18.2 ± 11.6 | -9.6 ± 10.5 | 9.9 ± 14.2 | 9.1 ± 13.9 |
| Percent weight change, % |  |  |  |  |  |
| Mean ± S.D | 10.1 ± 13.8 | 29.6 ± 19.9 | -11.8 ± 11.9 | 12.9 ± 17.6 | 14.6 ± 20.7 |
| Median [IQR] | 8.6 [1.7;16.9] | 26.0 [16.2;39.0] | -10.8 [-17.3;-5.3] | 10.5 [1.4;21.7] | 12.5 [1.6;25.0] |
| Delta BMI, kg/m2  Mean ± S.D | 1.4 ± 2.2 | 6.1 ± 3.7 | -4.3 ± 3.2 | 2.9 ± 4.8 | 2.7 ± 4.7 |
| Diagnosed with diabetes before cancer, number (%) | 473 (1) | 4,348 (9) | 231 (1) | 3,550 (7) | 8,602 (5) |
| Smoking, number (%) | 18,366 (38) | 19,542 (41) | 7,452 (47) | 20,937 (44) | 66,297 (42) |

Each participant in the high-to-lean BMI group was matched (1:3 ratio, without replacement) to participants from other trajectory groups based on sex, the time between BMI assessments (±1 year) and age at adult BMI measurement (±1 year).

Sociodemographic and BMI data at late adolescence were extracted from the Israel Defense Forces military pre-recruitment dataset. BMI and clinical data at adulthood were retrieved from the Maccabi Healthcare Service dataset. Country of origin was classified by the examinee’s father’s country of birth or by the grandfather’s country of birth if the father was born in Israel.*Unimpaired health was defined for people without any morbidities at pre-recruitment evaluation at adolescence that required medical follow-up or chronic treatment, and without a history of cancer or major surgery.

**Part B: A multivariable analysis of the matched cohort**

|  | **Lean-to-Lean BMI** | **Lean-to-High BMI** | **High-to-Lean BMI** | **High-to-High BMI** | **Total** |
| --- | --- | --- | --- | --- | --- |
| **Main analysis** | | | | | |
| Number of people | 421,865 | 261,643 | 16,065 | 84,447 | 784,020 |
| Diagnosed with obesity-related cancers | 2,235 | 3,076 | 60 | 601 | 5,972 |
| HR | 1 (reference) | 1.31 | 1.01 | 1.47 |  |
| 95% CI |  | 1.24 – 1.39 | 0.78 – 1.31 | 1.34 – 1.61 |  |
| P value |  | <0.001 | 0.919 | <0.001 |  |
| **Matched cohort** | | | | | |
| Number of people | 48,195 | 48,195 | 16,065 | 48,195 | 160,650 |
| Diagnosed with obesity-related cancers | 202 | 567 | 60 | 313 | 1,142 |
| HR | 1 (reference) | 1.32 | 0.98 | 1.37 |  |
| 95% CI |  | 1.10 – 1.59 | 0.73 – 1.30 | 1.15 – 1.64 |  |
| P value |  | 0.003 | 0.877 | <0.001 |  |

Associations between BMI trajectory and obesity-related cancer risk were consistent with the main findings.

Hazard ratios (HRs) and 95% confidence intervals (CIs) for obesity-related cancers according to BMI trajectory groups within each age stratum.

The main analysis is shown to facilitate comparison. The adjusted model was applied.

BMI, body mass index; HR, hazard ratio; CI, confidence interval.

**Supplementary Table S15: Defining BMI trajectories based on two time points in adulthood**

|  | **Lean-to-Lean BMI** | **Lean-to-High BMI** | **High-to-Lean BMI** | **High-to-High BMI** | **Total** |
| --- | --- | --- | --- | --- | --- |
| **Main analysis** | | | | | |
| Number of people | 421,865 | 261,643 | 16,065 | 84,447 | 784,020 |
| Diagnosed with obesity-related cancers | 2,235 | 3,076 | 60 | 601 | 5,972 |
| HR | 1 (reference) | 1.31 | 1.01 | 1.47 |  |
| 95% CI |  | 1.24 – 1.39 | 0.78 – 1.31 | 1.34 – 1.61 |  |
| P value |  | <0.001 | 0.919 | <0.001 |  |
| **With at least two adult BMI measurements** | | | | | |
| Total | 304,933 | 217,751 | 10,631 | 64,964 | 598,279 |
| A second adult BMI categorized as lean, number (%) | 257,533 (84.5) | 26,495 (12.2) | 6,691 (62.9) | 3,391 (5.2) |  |
| A second adult BMI categorized as high, number (%) | 47,400 (15.5) | 191,256 (87.8) | 3,940 (37.1) | 61,573 (94.8) |  |
| **The first and second adult BMI measurements are both categorized as lean, or both as high** | | | | | |
| Number of people | 252,831 | 185,765 | 6,584 | 60,396 | 505,576 |
| Diagnosed with obesity-related cancers | 1,353 | 2,266 | 30 | 481 | 4,130 |
| HR | 1 (reference) | 1.41 | 1.04 | 1.59 |  |
| 95% CI |  | 1.32 – 1.52 | 0.72 – 1.49 | 1.43 – 1.76 |  |
| P value |  | <0.001 | 0.850 | <0.001 |  |
| **Up to 1 BMI unit difference between the first and the second adult measurements** | | | | | |
| Number of people | 157,063 | 93,425 | 4,320 | 20,757 | 275,565 |
| Diagnosed with obesity-related cancers | 866 | 1,045 | 19 | 151 | 2,081 |
| HR | 1 (reference) | 1.36 | 1.00 | 1.46 |  |
| 95% CI |  | 1.23 – 1.49 | 0.64 – 1.58 | 1.23 – 1.74 |  |
| P value |  | <0.001 | 0.986 | <0.001 |  |

Note that among 800,024 people, 598,279 had a second adult BMI measurement (75% of the study cohort). To reduce potential misclassification, an additional adult BMI measurement measured at a routine clinical visit was used to limit the analysis to those with persistent adult BMI: (i) including only people with both the first and second adult BMI categorized as lean, or both as high; (ii) including only people with up to one BMI unit difference between the first and the second adult measurements.

The main analysis is shown to facilitate comparison. The adjusted model was applied.

BMI, body mass index; HR, hazard ratio; CI, confidence interval

**Supplementary Table S16: Stringently controlling for baseline BMI**

| **High-to-lean BMI vs. High-to-high BMI** | | |
| --- | --- | --- |
| **Adjusted model** | |  |
| HR | | 0.70 |
| 95% CI | | 0.54 – 0.93 |
| P value | | 0.012 |
| **Adjusted model + adolescent BMI** | |  |
| HR | | 0.71 |
| 95% CI | | 0.54 – 0.93 |
| P value | | 0.014 |
| **High-to-lean BMI vs. Matched cohort** | | |
| **Adjusted model** | |  |
| HR | | 0.71 |
| 95% CI | | 0.53 – 0.94 |
| P value | | 0.019 |
| **Adjusted model + adolescent BMI** | |  |
| HR | | 0.71 |
| 95% CI | | 0.53 – 0.95 |
| P value | | 0.019 |
|  | **Matched cohort** | **High-to-lean BMI** |
| Number of people | 48,240 | 16,080 |
| Women, % | 54 | 54 |
| **At adolescence** |  |  |
| BMI |  |  |
| Mean ± S.D | 27.8 ± 2.5 | 27.5 ± 2.7 |
| Median [IQR] | 27.1 [26.1; 28.7] | 26.6 [25.8; 28.1] |
| **At adulthood** |  |  |
| Age at the adult measurement of BMI | 28.7 ± 8.0 | 29.3 ± 8.6 |
| Adult BMI |  |  |
| Mean ± S.D | 30.8 ± 4.6 | 23.1 ± 1.6 |
| Median [IQR] | 29.8 [27.4; 33.1] | 23.5 [22.3; 24.3] |
| Delta weight |  |  |
| Kg | 10.1 ± 13.0 | -9.7 ± 10.6 |
| Percent, % | 13.4 ± 16.8 | -11.8 ± 11.9 |
| Delta BMI, kg/m2 | 3.0 ± 4.4 | -4.4 ± 3.3 |
| Unimpaired health at adolescence, % | 68 | 69 |
| Diagnosed with diabetes before cancer, % | 6 | 2 |
| Smoking, % | 43 | 47 |

We established a control group by matching people with high-to-high BMI for sex, birth year (within 5 years), and adolescent BMI (within 3 units) to people with high-to-lean BMI (3:1 ratio; n = 48,240). This matching aimed to minimize residual confounding by adolescent BMI, which could remain even after adjustment of Cox models. Note that 203 of 16,283 people from the high-to-lean BMI group were not able to be matched to three people according to the matching criteria, and were thus excluded.

BMI, body mass index; HR, hazard ratio; CI, confidence interval; S.D, standard deviation; IQR, interquartile range

**Supplementary Table S17: Sensitivity analysis with follow-up starting at the adolescent BMI measurement**

|  | **Lean-to-Lean BMI** | **Lean-to-High BMI** | **High-to-Lean BMI** | **High-to-High BMI** | **Total** |
| --- | --- | --- | --- | --- | --- |
| **Main analysis** | | | | | |
| Number of people | 421,865 | 261,643 | 16,065 | 84,447 | 784,020 |
| Diagnosed with obesity-related cancers | 2,235 | 3,076 | 60 | 601 | 5,972 |
| HR | 1 (reference) | 1.31 | 1.01 | 1.47 |  |
| 95% CI |  | 1.24 – 1.39 | 0.78 – 1.31 | 1.34 – 1.61 |  |
| P value |  | <0.001 | 0.919 | <0.001 |  |
| **Follow -up from adolescent BMI measurement** |  |  |  |  |  |
| Number of people | 421,865 | 261,643 | 16,065 | 84,447 | 784,020 |
| Diagnosed with obesity-related cancers | 2,235 | 3,076 | 60 | 601 | 5,972 |
| HR | 1 (reference) | 1.27 | 1.03 | 1.44 |  |
| 95% CI |  | 1.20 – 1.34 | 0.79 – 1.33 | 1.31 – 1.57 |  |
| P value |  | <0.001 | 0.847 | <0.001 |  |

Model was repeated with follow-up starting at the first (adolescent) BMI measurement, testing time zero as the hypothetical intervention.

The main analysis is shown to facilitate comparison. The adjusted model was applied.

BMI, body mass index; HR, hazard ratio; CI, confidence interval

**Supplementary Figure S2: A spline model across adulthood-adolescent BMI difference**

**
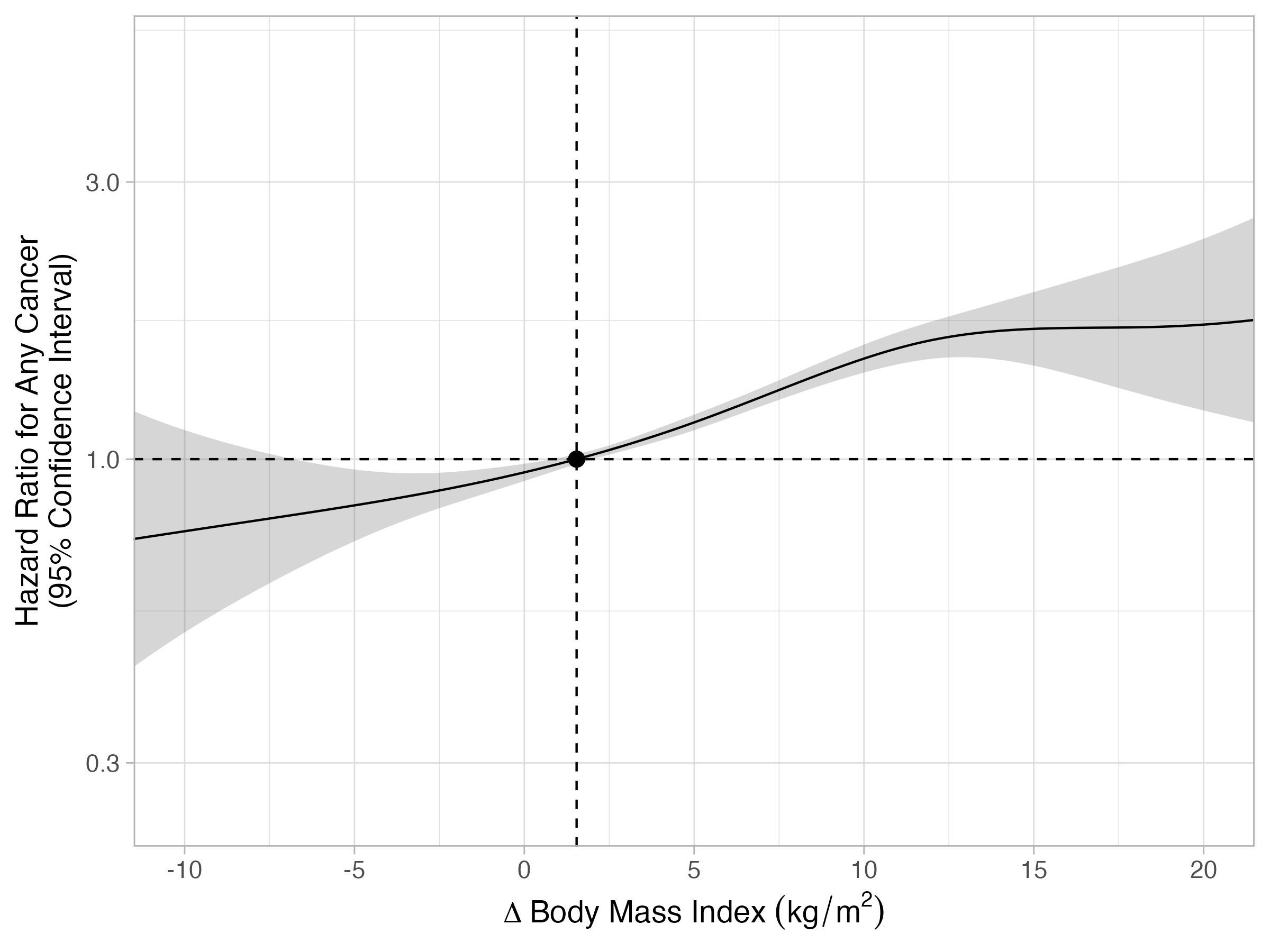
**

**A spline model of the association between the adolescent-adulthood difference in BMI and obesity-related cancers.** The spline model was derived for the hazard ratio between the adulthood-adolescence difference in BMI and incident obesity-related cancers. The adjusted model was applied, with the addition of adolescent BMI. The median BMI difference for the lean-to-lean BMI group was used as a reference (1.54 kg/m^2^). The lines indicate the hazard ratios, and the shading indicates 95% confidence intervals.

**Supplementary Table S18: A linear adjusted Cox model using adolescent BMI and adolescent-to-adulthood delta BMI as continuous variables**

| **Cancer type** | **Adolescent BMI** | **Adolescent-to-adulthood Δ BMI** |
| --- | --- | --- |
|  | **(per 1 unit of BMI increment)** | **(per 1 unit of BMI increment)** |
| **Positively associated with both adolescent BMI and Adolescent-to-adulthood Δ BMI** | | |
| Obesity-related cancers (IARC)* | 1.03 | 1.03 |
|  | 1.02 - 1.04 | 1.03 - 1.04 |
|  | <0.001 | <0.001 |
| Colorectal* | 1.03 | 1.03 |
|  | 1.01 – 1.05 | 1.02 – 1.04 |
|  | 0.001 | <0.001 |
| Kidney* | 1.07 | 1.06 |
|  | 1.04 – 1.10 | 1.04 – 1.08 |
|  | <0.001 | <0.001 |
| Pancreas* | 1.11 | 1.04 |
|  | 1.07 – 1.15 | 1.01 – 1.07 |
|  | <0.001 | 0.004 |
| Stomach* | 1.05 | 1.05 |
|  | 1.00 – 1.10 | 1.02 – 1.09 |
|  | 0.036 | 0.002 |
| Thyroid* | 1.04 | 1.04 |
|  | 1.02 – 1.05 | 1.03 – 1.06 |
|  | <0.001 | <0.001 |
| Uterus* | 1.08 | 1.10 |
|  | 1.04 – 1.12 | 1.08 – 1.12 |
|  | <0.001 | <0.001 |
| Hodgkin's lymphoma | 1.04 | 1.04 |
|  | 1.00 – 1.08 | 1.01 – 1.07 |
|  | 0.031 | 0.025 |
| **Positively associated with adolescent BMI but not associated with adolescent-to-adulthood Δ BMI** | | |
| Brain | 1.04 | 1.02 |
|  | 1.01 – 1.08 | 1.00 – 1.05 |
|  | 0.012 | 0.114 |
| Non-Hodgkin's lymphoma | 1.03 | 1.00 |
|  | 1.01 – 1.05 | 0.99 – 1.02 |
|  | 0.001 | 0.666 |
| Leukemia | 1.06 | 0.99 |
|  | 1.03 – 1.09 | 0.97 – 1.01 |
|  | <0.001 | 0.479 |
| **Not associated with adolescent BMI but positively associated with adolescent-to-adulthood Δ BMI** | | |
| Esophagus* | 1.05 | 1.08 |
|  | 0.95 - 1.16 | 1.01 - 1.14 |
|  | 0.345 | 0.015 |
| Oral | 1.02 | 1.06 |
|  | 0.95 - 1.09 | 1.02 - 1.11 |
|  | 0.587 | 0.009 |
| **Negatively associated with adolescent BMI and positively associated with adolescent-to-adulthood Δ BMI** | | |
| Breast (postmenopausal)* | 0.98 | 1.03 |
|  | 0.96 - 1.00 | 1.02 - 1.04 |
|  | 0.039 | <0.001 |
| **Negatively associated with both adolescent BMI and adolescent-to-adulthood Δ BMI** | | |
| Cervix | 0.98 | 0.96 |
|  | 0.97 – 0.99 | 0.95 – 0.97 |
|  | <0.001 | <0.001 |
| **Not associated with adolescent BMI but negatively associated with adolescent-to-adulthood Δ BMI** | | |
| Lung | 0.99 | 0.96 |
|  | 0.96 – 1.02 | 0.94 – 0.98 |
|  | 0.364 | <0.001 |
| **Not associated with adolescent BMI or with adolescent-to-adulthood Δ BMI** | | |
| Multiple Myeloma* | 1.03 | 1.01 |
|  | 1.00 - 1.07 | 0.99 - 1.04 |
|  | 0.083 | 0.415 |
| Ovary* | 1.05 | 1.03 |
|  | 0.99 – 1.11 | 0.99 – 1.07 |
|  | 0.086 | 0.158 |
| Melanoma | 1.01 | 1.00 |
|  | 1.00 – 1.03 | 0.99 – 1.01 |
|  | 0.063 | 0.51 |
| Testis | 1.01 | 1.03 |
|  | 0.98 – 1.05 | 0.99 – 1.07 |
|  | 0.541 | 0.102 |
| Liver and Biliary * | 1.04 | 0.99 |
|  | 0.98 - 1.11 | 0.94 - 1.04 |
|  | 0.198 | 0.643 |
| Prostate | 0.99 | 1.01 |
|  | 0.97 – 1.01 | 0.99 – 1.02 |
|  | 0.39 | 0.22 |

The Cox proportional hazard model was applied in the analyses of adolescent BMI (continuous) and adolescent-to-adulthood delta BMI (continuous), and other variables were included in the main analysis. Adjusted hazard ratios (HRs) of the adolescent BMI component (per 1 unit increment) and of the adolescent-to-adulthood delta BMI (per 1 unit increment) are shown to facilitate the comparisons. For each type of cancer, the first row presents the HRs, the second one presents the 95% confidence interval, and the third one presents the P-values. *Obesity-related cancers were determined according to the International Agency for Research on Cancer (IARC), and encompass 11 specific cancer types: esophagus, postmenopausal breast, liver and gallbladder, stomach, pancreas, colon and rectum, kidney, multiple myeloma, thyroid, uterus, and ovary.

Hazard ratio HR cell colors indicate the direction of the association and whether it is statistically significant, as follows: HR >1 and P-value <0.05; HR >1 and P-value ≥0.05; HR <1 and P-value <0.05; HR <1 and P-value ≥0.05.

**Supplementary Table S19: A linear adjusted model of each 5-kg weight gain from initial body weight at adolescence**

|  |  | **Risk for obesity-related cancers** |
| --- | --- | --- |
| For each 5kg weight gain (our cohort) | HR | 1.05 |
|  | 95% CI | 1.04 – 1.06 |
| For each 5kg weight gain  (Zheng, JAMA, 2017) | HR | 1.06 |
|  | 95% CI | 1.02 – 1.09 |

To facilitate comparison with other cohorts in this field, we conducted an additional linear analysis to assess the risk of obesity-related cancers with each 5-kg increase from the initial body weight at adolescence and the risk for obesity-related cancers. Our findings revealed similar point estimates to those previously reported by Zheng et al.. ^26^

1. Lauby-Secretan, B., Scoccianti, C., Loomis, D., Grosse, Y., Bianchini, F., & Straif, K. (2016). Body fatness and cancer—viewpoint of the IARC Working Group. *New England journal of medicine*, *375*(8), 794-798.‏ [↑](#footnote-ref-2)
